# Supplementary material for: A Multi-Faceted Analysis Showing CRNDE Transcripts and a Recently Confirmed Micropeptide as Important Players in Ovarian Carcinogenesis
Source: Int J Mol Sci. 2024 Apr 16;25(8):4381. doi: 10.3390/ijms25084381 (PMC11050281; doi:10.3390/ijms25084381)
Supplement: Supplementary file 1 [file ijms-25-04381-s001.zip › ijms-2946734-supplementary.pdf]

Supplementary Materials for

Multi-faceted analysis showing *CRNDE* transcripts and the lately confirmed micropeptide as important players in ovarian carcinogenesis

Anna Balcerak *et al.*

Corresponding author’s email: lukszafron@gmail.com

Supplementary Text

Mass spectrometry-based identification of the CRNDEP micropeptide

1. **Immunoprecipitation (IP), IP sample preparation, protein digestion and peptide fractionation**

   The IP experiment was conducted according to Thermo recommendations, using 10 ml of a protein lysate (conc. 11.6 mg/ml), generated with RIPA+inhibitors, from one high-grade ovarian carcinoma (hgOvCa) sample characterized by high CRNDEP expression. Based on our dot blot analysis, utilizing a standard curve made of a synthetic CRNDEP epitope (in known concentrations), earlier used for the anti-CRNDEP antibody development, we estimated that the given amount of the lysate should contain approximately 10 nanomoles of CRNDEP. Apart from the lysate, 240 µg of the anti-CRNDEP antibody and 640 µl of the Dynabeads Protein A (Thermo) magnetic resin were used in this IP experiment. CRNDEP was then eluted from the resin with 0.1 M glycine, pH = 2 followed by instant neutralization of the sample with 1M TRIS buffer, pH = 8.5, which gave the final IP eluate in the total amount of 3.3 ml. 1.98 ml of this eluate (corresponding to about 6 nanomoles of CRNDEP) were used as a sample for the mass spectrometry analysis. This sample was buffered with 100 mM triethylamonium bicarbonate (TEAB) and supplemented with 5% trifluoroethanol. Cysteine bridges were reduced by 1 hour incubation with 10 mM tris(2-carboxyethyl)phosphine (TCEP) at 37°C followed by the reaction with 25 mM s-methylmethanethiosulfonate (MMTS). Proteins were transferred onto three Vivacon 30 kDa cut-off filters (Sartorius AG, Göttingen, Germany) and spun at 14,500 g for 30 min to remove high-molecular-mass proteins. An additional step of elution, with 0.5 M NaCl, was performed to improve recovery. Combined eluates were digested overnight at 37 °C with 4 µg of trypsin (Promega). Samples were dried and then resuspended in 10 mM ammonium hydroxide (AH). Afterward, peptides were fractionated using high-pH reverse-phase chromatography on eight C18 Evotip trap columns (Evosep Biosystems, Odense, Denmark). Evotips were activated with 25 µl of 0.1% formic acid (FA) in acetonitrile (ACN) by 1 min centrifugation at 600 g followed by 2 min incubation in 2-propanol. After equilibration with 100 µl of 10 mM AH in water, the peptide solution was loaded onto the solid phase. The elution was carried out in rising ACN concentrations in the presence of 10 mM AH. Five fractions were collected using the following solutions: 5% ACN, 10% ACN, 15% ACN, 40% ACN and 100% ACN. Peptides were then dried in Speedvac overnight and reconstituted in the liquid chromatography – mass spectrometry (LC-MS) phase A (0.1% FA in water).

2. **LC-MS analysis**

   The peptide fractions were analyzed using LC-MS system composed of Evosep One (Evosep Biosystems) coupled with the Orbitrap Exploris 480 mass spectrometer (Thermo) via Flex ion source (Thermo). Samples were loaded onto disposable Evotips C18 trap columns according to the manufacturer's protocol. Chromatography was carried out at a 220 nl/min flow rate using the 88 min (15 samples per day) preformed gradient on the EV1106 analytical column (Dr Maisch C18 AQ, 1.9 µm beads, 150 µm ID, 15 cm long, Evosep Biosystems). MS data were acquired in positive mode with a data-dependent method (DDA) using the following ion optics parameters: HCD normalized

collision energy of 30%, spray voltage set to 2.1 kV, funnel RF level set to 40, and a heated capillary temperature set to 275 °C. DDA Cycle time was 1 s. Internal Mass Calibration by EASY-IC was used in the Run Start mode. The MS1 resolution was set to 120,000 with a 500% normalized AGC target, Auto maximum inject time and a scan range of 200 to 2,000 m/z. Preferably the ions with the maximum charge of 6, m/z within the range of 200-2,000 and corresponding to 5-40 aa length tryptic peptides derived from CRNDEP with the maximum of 2 missed cleavages and Methylthio (C) modification were subjected to fragmentation. Inclusion list Mass tolerance was set to 10 ppm with the option of a dependent scan on other most intense ions set to off. For MS2, the resolution was set to 30,000 with a 1,000% normalized AGC target and Auto maximum inject time.

3. MS data processing

The acquired MS/MS raw data files were processed to peak lists with Mascot Distiller (v. 2.5.1, Boston, MA, USA) and submitted to the Mascot search engine (v. 2.5.1, Matrix Science). The database was composed of SwissProt *Homo sapiens* entries, and contaminant proteins retrieved from the common Repository of Adventitious Proteins. The search parameters were as follows: enzyme specificity – semitrypsin; number of missed cleavages – 2; parent and fragment ions mass error tolerances – 10 ppm and 0.1 Da, respectively; fixed modification – Methylthio C; variable modification – Oxidation M. The statistical significance of identifications was determined using a target/decoy database approach and a procedure that provided q-value estimates for each peptide spectrum match (PSM) in the data set. Only PSMs with q-values ≤ 0.01 were regarded as confidently identified. All the peptide sequences matched to database entries representing contaminant proteins were rejected. Proteins represented by less than two peptides were excluded from further analysis. Proteins identified by a subset of peptides from other proteins were filtered out from the results, and those matching the same set of peptides were grouped together into metaproteins. Mascot results postprocessing was performed using the MScan app, available at http://proteom.ibb.waw.pl/mscan.

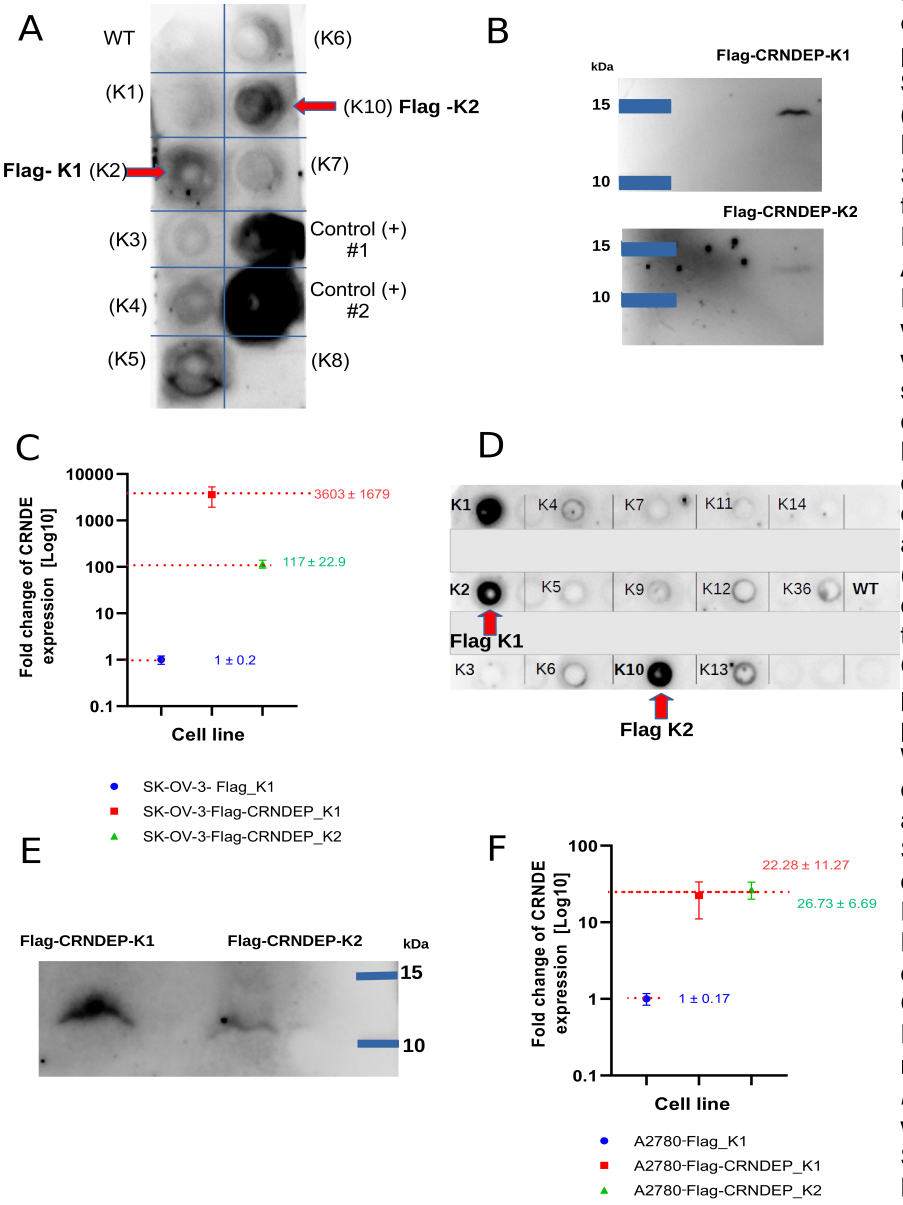

Fig. S1. Analysis of Flag-tag or CRNDEP-Flag fusion protein stable expression in SK-OV-3 (A,B,C) and A2780 (D,E,F) cells using the anti-Flag antibody. A: Daughter SK-OV-3 cell line clones with the highest expression of the Flag tag integrated into the AAVS1 site (Flag-K1(K2) and Flag-K2(K10)) are marked with red arrows. The K5 clone was morphologically less similar to the mother SK-OV-3 cells than the daughter Flag-K1(K2) and Flag-K2(K10) clones, therefore it was excluded from further analyses. As a positive control (+), a protein lysate from HeLa cells ectopically expressing the Flag tag fused to CRNDEP, encoded by the pCR3-2xFLAG-CRNDEP plasmid, was used. **B:** Western blot analysis for two clones (Flag-CRNDEP_K1, and Flag-CRNDEP_K2) of the SK-OV-3 cell line with stable expression of the CRNDEP-Flag fusion protein. **C:** The RT-qPCR analysis of *CRNDE* expression in SK-OV-3-Flag-CRNDEP_K1 and SK-OV-3-Flag-CRNDEP_K2 clones. As reference genes, *HGPRT* an *PPIA* were used. The results were calibrated to the control SK-OV-3-Flag_K1 clone. **D:** Daughter A2780 cell line clones with the highest expression of the Flag tag integrated into the AAVS1 site (Flag-K1(K2) and Flag-K2(K10)) are marked with red arrows. The K1 clone was morphologically less similar to mother A2780 cells than the daughter Flag-K1(K2) and Flag-K2(K10) clones, therefore it was excluded from further analyses. **E:** Western blot analysis for two clones (Flag-CRNDEP_K1, and Flag-CRNDEP_K2) of the A2780 cell line with stable expression of the CRNDEP-Flag fusion protein. **F:** RT-qPCR analysis of *CRNDE* expression in A2780 Flag-CRNDEP_K1 and Flag-CRNDEP_K2 clones. As reference genes, *HGPRT* an *PPIA* were used. The results were calibrated to the control A2780-Flag_K1 clone. AAVS1: Adeno-Associated Virus Integration Site 1.

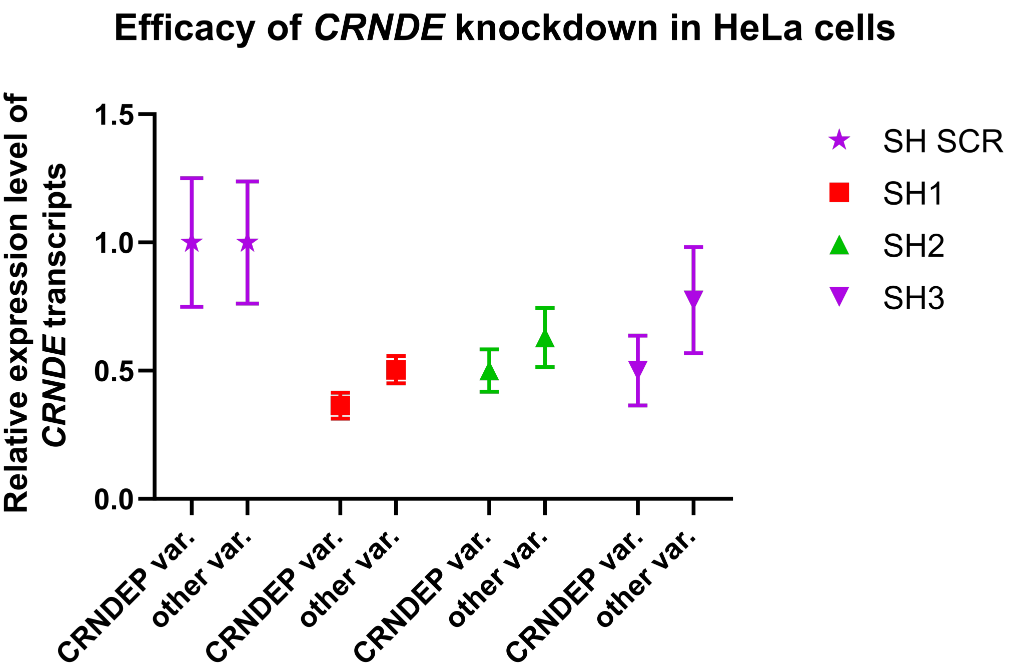

Fig. S2. Transient silencing of different *CRNDE* transcripts in HeLa cells. Three shRNAs (SH1, SH2 and SH3) were used for *CRNDE* silencing. Verification of *CRNDE* transcripts' expression was performed using RT-qPCR. As references, the *HGPRT* and *PPIA* genes were used. Results obtained for the three CRNDE-silencing shRNAs were calibrated to the expression of *CRNDE* transcripts obtained for the HeLa cell line transiently expressing the control shRNA, SH SCR (scrambled). CRNDEP var. – CRNDEP-coding transcript; other var. – other *CRNDE* transcripts.

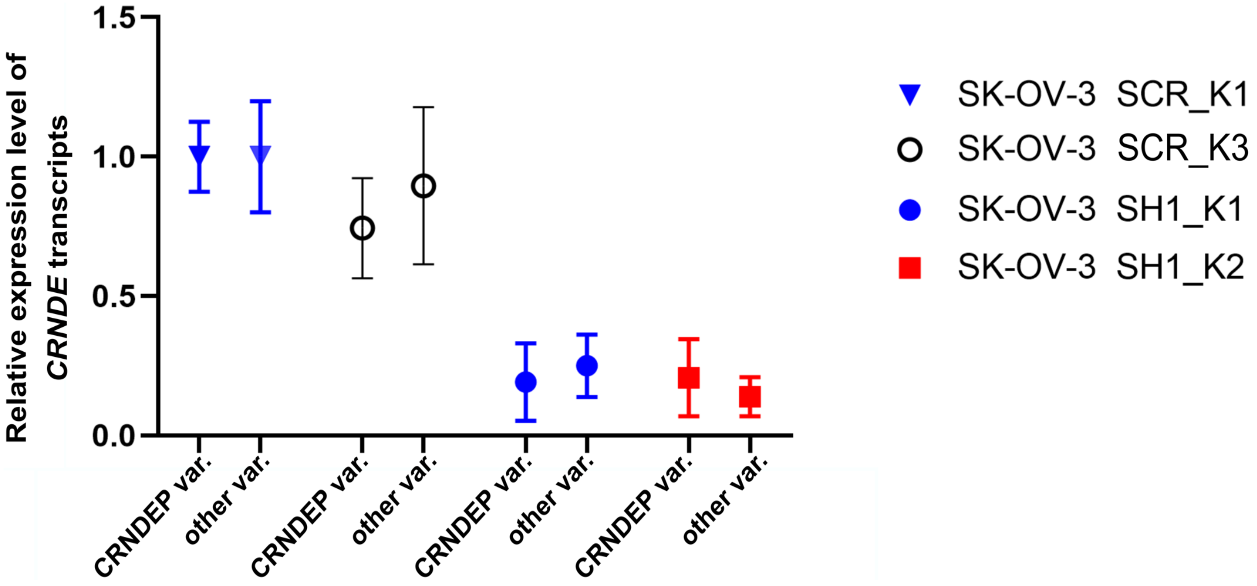

Fig. S3. *CRNDE* transcripts' expression in SK-OV-3 cells with stable *CRNDE* silencing. Expression of *CRNDE* transcripts was assessed by RT-qPCR. As references, *HGPRT* and *PPIA* genes were used. The results were calibrated to the expression of *CRNDE* transcripts obtained for the SK-OV-3 cell line stably expressing the control shRNA, SCR_K1. CRNDEP var. – CRNDEP-coding transcript; other var. – other *CRNDE* transcripts.

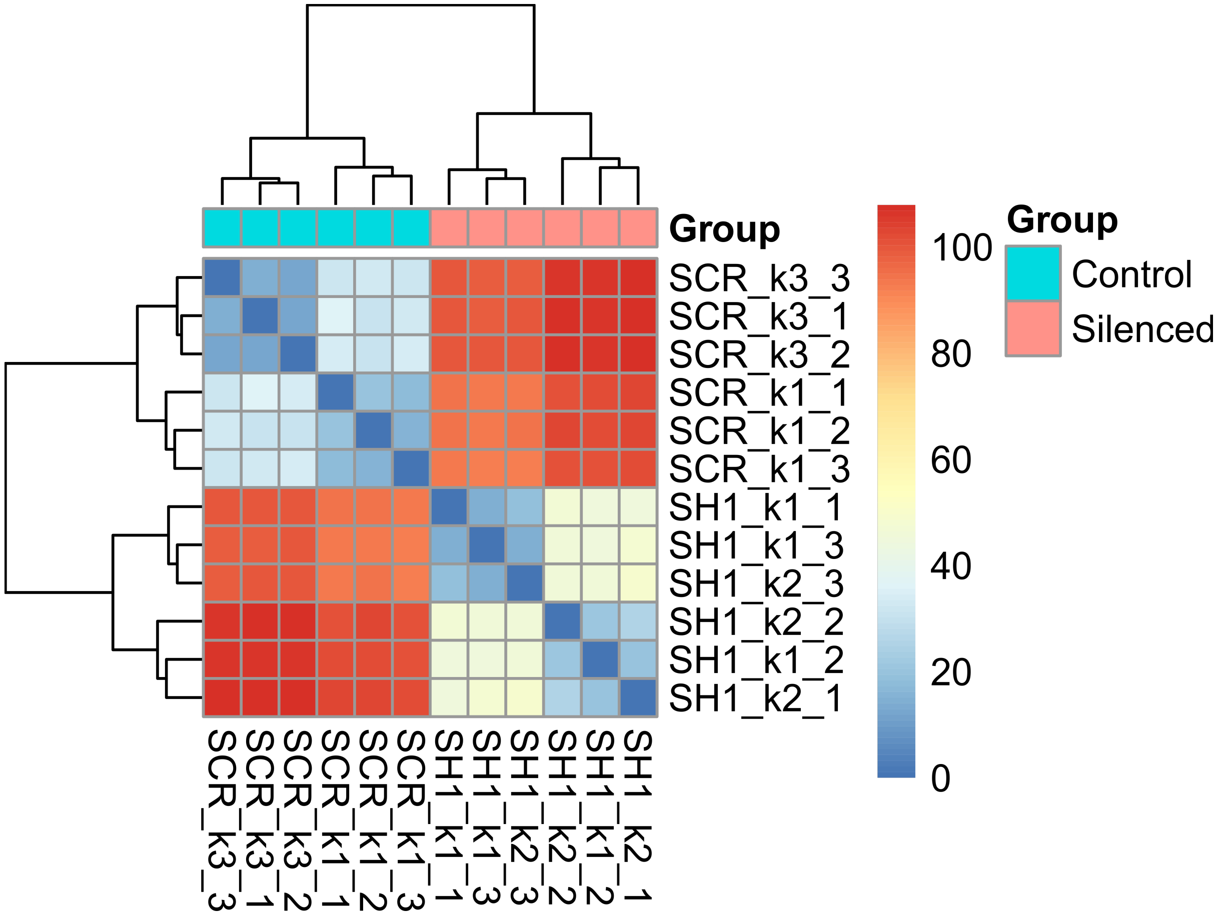

Fig. S4. Overall transcriptomic differences between SK-OV-3 cells with (clones SH1_k1 and SH1_k2) and without (clones SCR_k1 and SCR_k3) *CRNDE* silencing. The distances in expression patterns between the samples are displayed here as a heatmap with hierarchical clustering. The analysis revealed great similarity in expression profiles between the SK-OV-3 cells with *CRNDE* knockdown and also between the control SK-OV-3 cells. By contrast, the expression differences between the silenced (SK-OV-3-SH1) and the control (SK-OV-3-SCR) cells were clearly visible.

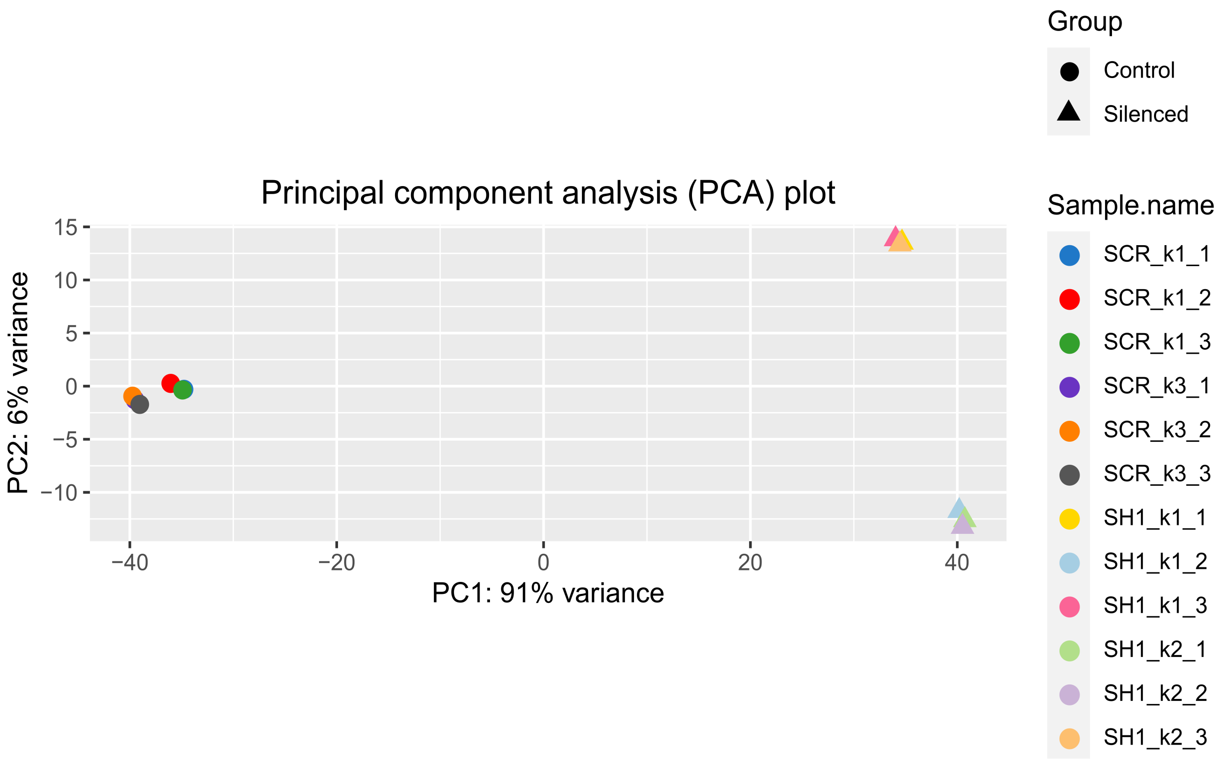

Fig. S5. Principal Component Analysis (PCA) of the RNA-seq data obtained for SK-OV-3 cells with (clones SH1_k1 and SH1_k2) and without (clones SCR_k1 and SCR_k3) *CRNDE* silencing. The differences between the cells with *CRNDE* silencing and the control cells accounted for 91% of the total gene expression variance in this sample set (PC1).

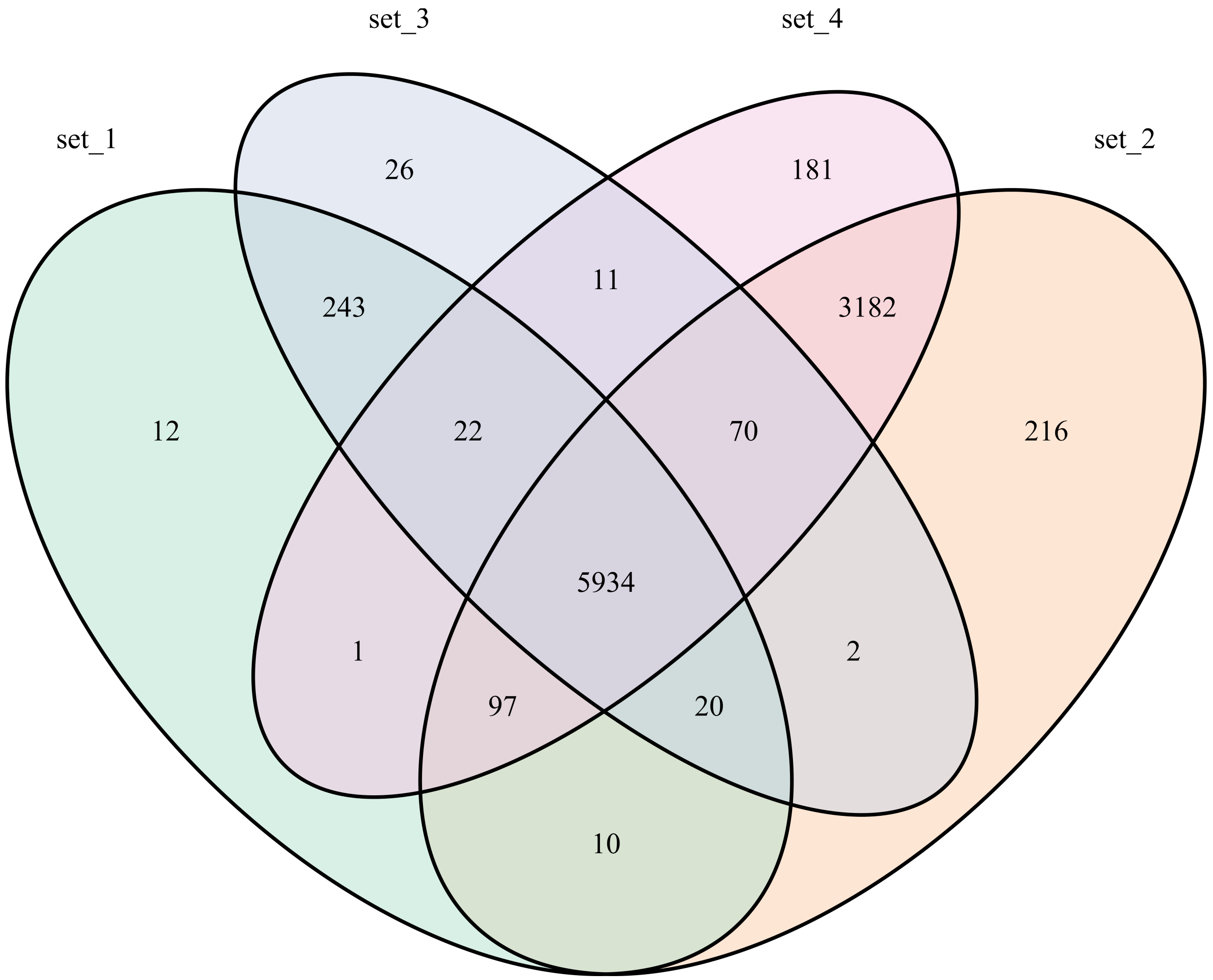

Fig. S6. Venn diagram depicting similarity of four gene sets, comprising genes with significantly altered expression after *CRNDE* silencing. These gene sets were obtained by using different combinations of sequence aligning (HISAT2, STAR) and expression analyzing (DESeq2, edgeR) programs. Set_1: HISAT2 and DESeq2, Set_2: HISAT2 and edgeR, Set_3: STAR and DESeq2, Set_4: STAR and edgeR.

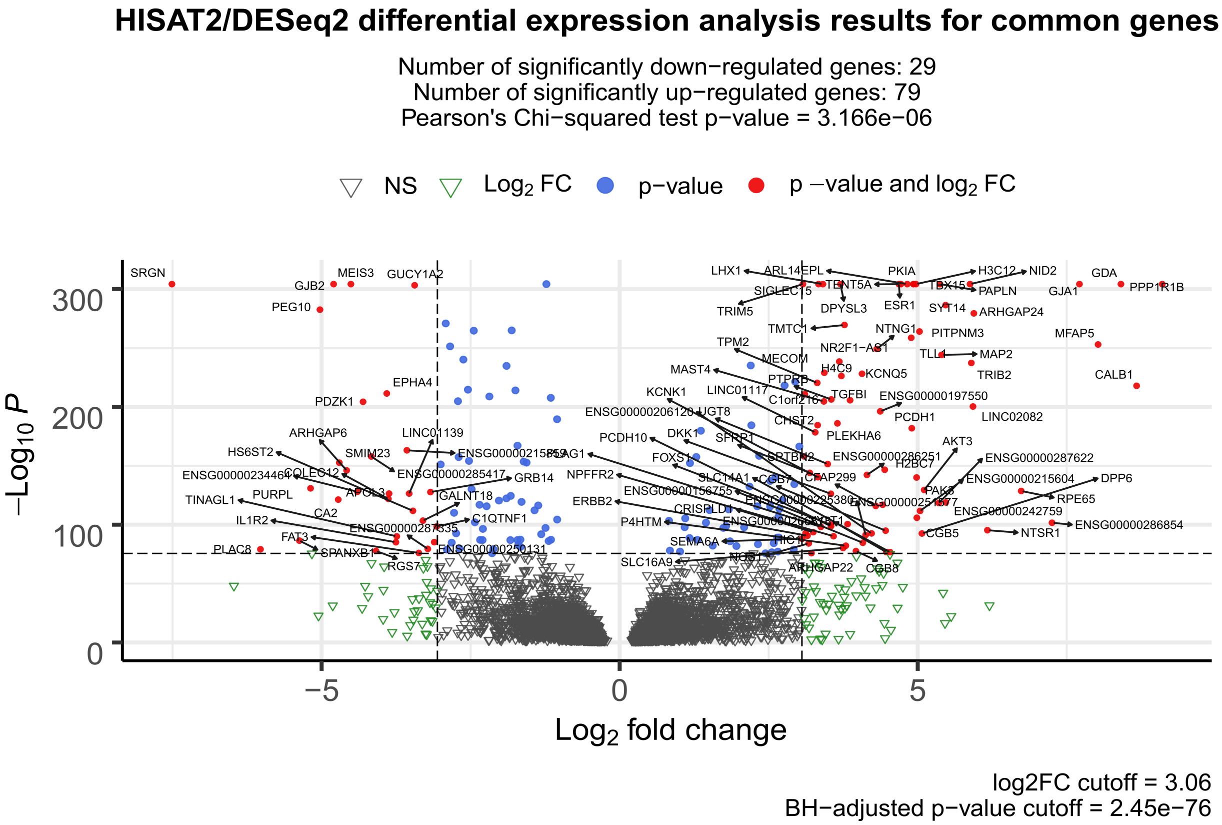

Fig. S7. Volcano plot for common set of 5934 differentially expressed genes (using p-values and FC values obtained with the HISAT2 and DESeq2 programs). Blue dots represent genes that meet only the Benjamini-Hochberg-adjusted p-value cutoff (2.45E-76), green triangles denote genes meeting the $|\log_2 FC|$ cutoff of 3.06. Genes passing both filters (red dots) have their names displayed on the plot. The plot is additionally supplemented with numbers of significantly up- and down-regulated genes, as well as the Pearson's Chi-squared test result.

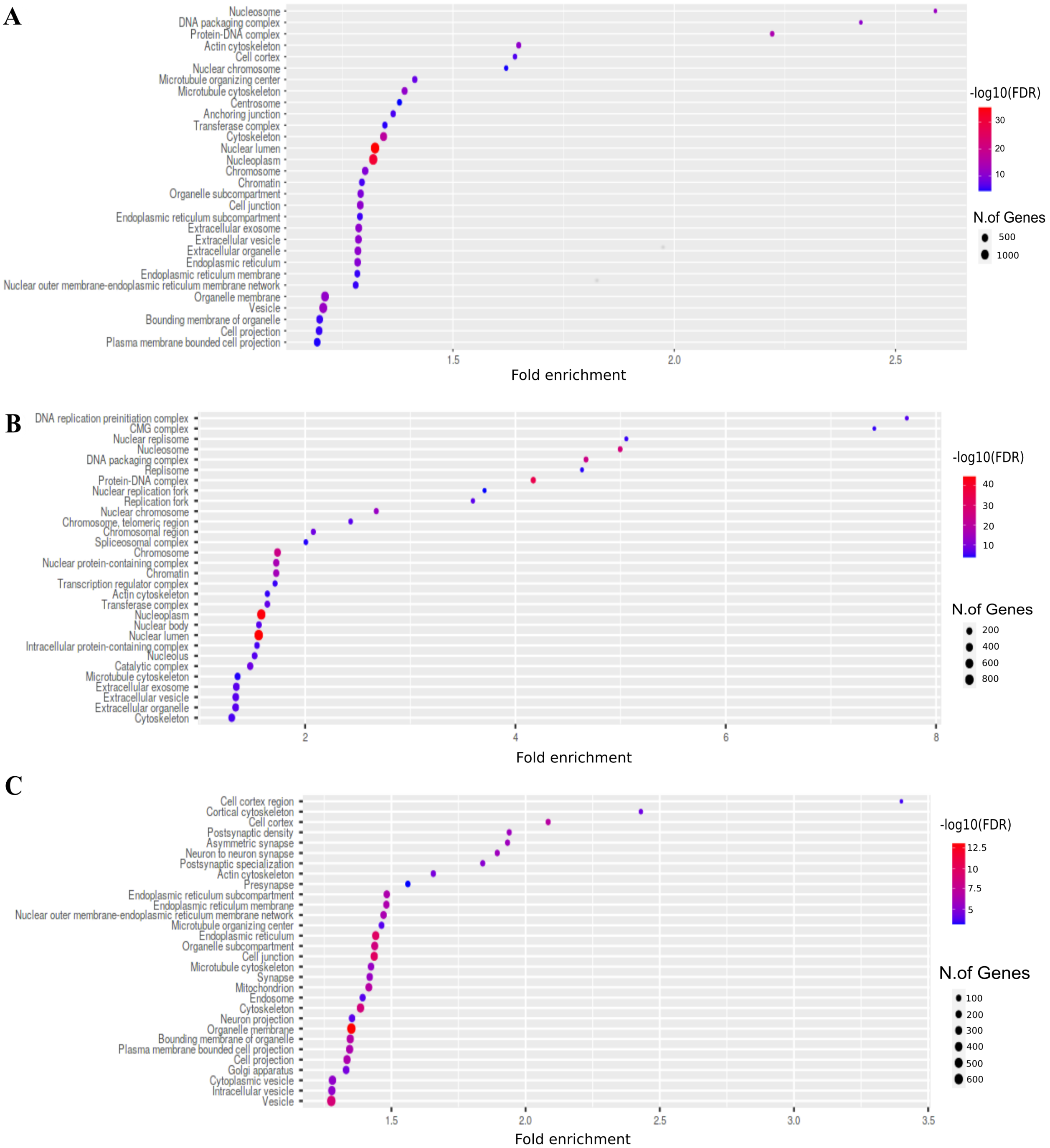

Fig. S8. Gene ontology analysis (cellular components, CC) performed for our RNA-seq data with the ShinyGo app and the GO database. All graphs represent ontological terms most enriched in genes, expression of which was significantly altered after *CRNDE* silencing. In all the graphs, terms are sorted according to the decreasing fold enrichment values. **A:** terms enriched in genes expression of which was both increased or decreased (FC ≤ 0.8 or FC ≥ 1.2) after *CRNDE* silencing; **B:** terms enriched in genes with increased expression after *CRNDE* silencing (FC ≥ 1.2); **C:** terms enriched in genes with decreased expression after *CRNDE* silencing (FC ≤ 0.8), when compared to the control cell lines.

Table S1. Gene ontology analysis (CC) performed for our RNA-seq data with the EnrichR app and the GO database.

A

Cellular component FC ≤ 0.8 or FC ≥ 1.2				
#	Ontological terms	Ratio of identified genes in each category	Percentage of identified genes in each category	Adj. p-value
1.	cytoskeleton (GO:0005856)	226/600	37.7%	1.7E-07
2.	intracellular membrane-bounded organelle (GO:0043231)	1540/5192	29.7%	1.7E-07
3.	nucleus (GO:0005634)	1321/4484	29.5%	2.1E-05
4.	actin cytoskeleton (GO:0015629)	117/316	37.0%	2.5E-03
5.	endosome membrane (GO:0010008)	118/325	36.3%	4.9E-03
6.	endoplasmic reticulum membrane (GO:0005789)	231/712	32.4%	1.3E-02
7.	focal adhesion (GO:0005925)	133/387	34.4%	1.9E-02
8.	cell-substrate junction (GO:0030055)	135/394	34.3%	1.9E-02
9.	CMG complex (GO:0071162)	8/10	80.0%	3.3E-02
10.	intracellular organelle lumen (GO:0070013)	264/848	31.1%	4.9E-02

B

Cellular component FC ≥ 1.2				
#	Ontological terms	Ratio of identified genes in each category	Percentage of identified genes in each category	Adj. p-value
1.	nucleus (GO:0005634)	766/4484	17.1%	2.18E-14
2.	intracellular membrane-bounded organelle (GO:0043231)	866/5192	16.7%	2.28E-14
3.	intracellular non-membrane-bounded organelle (GO:0043232)	218/1158	18.8%	3.77E-06
4.	nuclear lumen (GO:0031981)	151/745	20.3%	4.26E-06
5.	nucleolus (GO:0005730)	143/733	19.5%	7.96E-05
6.	CMG complex (GO:0071162)	8/10	80.0%	2.30E-04
7.	chromosome (GO:0005694)	42/160	26.3%	4.82E-04
8.	alpha DNA polymerase:primase complex (GO:0005658)	5/5	100.0%	2.12E-03
9.	cytoskeleton (GO:0005856)	113/600	18.8%	2.89E-03
10.	Golgi medial cisterna (GO:0005797)	7/11	63.6%	6.00E-03
11.	nuclear chromosome (GO:0000228)	23/83	27.7%	1.39E-02
12.	actin cytoskeleton (GO:0015629)	63/316	19.9%	1.92E-02
13.	focal adhesion (GO:0005925)	73/387	18.9%	3.45E-02
14.	cell-substrate junction (GO:0030055)	74/394	18.8%	3.45E-02
15.	ESC/E(Z) complex (GO:0035098)	5/8	62.5%	4.45E-02

C

Cellular component FC ≤ 0.8				
#	Ontological terms	Ratio of identified genes in each category	Percentage of identified genes in each category	Adj. p-value
1.	endoplasmic reticulum membrane (GO:0005789)	136/712	19.10%	6.81E-03
2.	endosome membrane (GO:0010008)	69/325	21.23%	1.60E-02
3.	postsynaptic density (GO:0014069)	35/138	25.36%	1.60E-02
4.	asymmetric synapse (GO:0032279)	34/133	25.56%	1.60E-02
5.	lysosome (GO:0005764)	90/477	18.87%	5.28E-02
6.	cytoskeleton (GO:0005856)	109/600	18.17%	5.29E-02
7.	spermatoproteasome complex (GO:1990111)	4/5	80.00%	9.01E-02
8.	endoplasmic reticulum tubular network (GO:0071782)	9/23	39.13%	1.01E-01
9.	early endosome membrane (GO:0031901)	24/97	24.74%	1.01E-01
10.	intrinsic component of mitochondrial membrane (GO:0098573)	6/12	50.00%	1.06E-01

Ontological terms most enriched in genes, expression of which was significantly altered after *CRNDE* silencing, are sorted according to the increasing FDR-adjusted p-value. In table **A**, terms most enriched in all differentially expressed genes (up- and downregulated) are shown; Tables **B** and **C** include terms most enriched in up-regulated genes (FC ≥ 1.2) and down-regulated genes (FC ≤ 0.8), respectively, when compared to the control cells. FC – fold change.

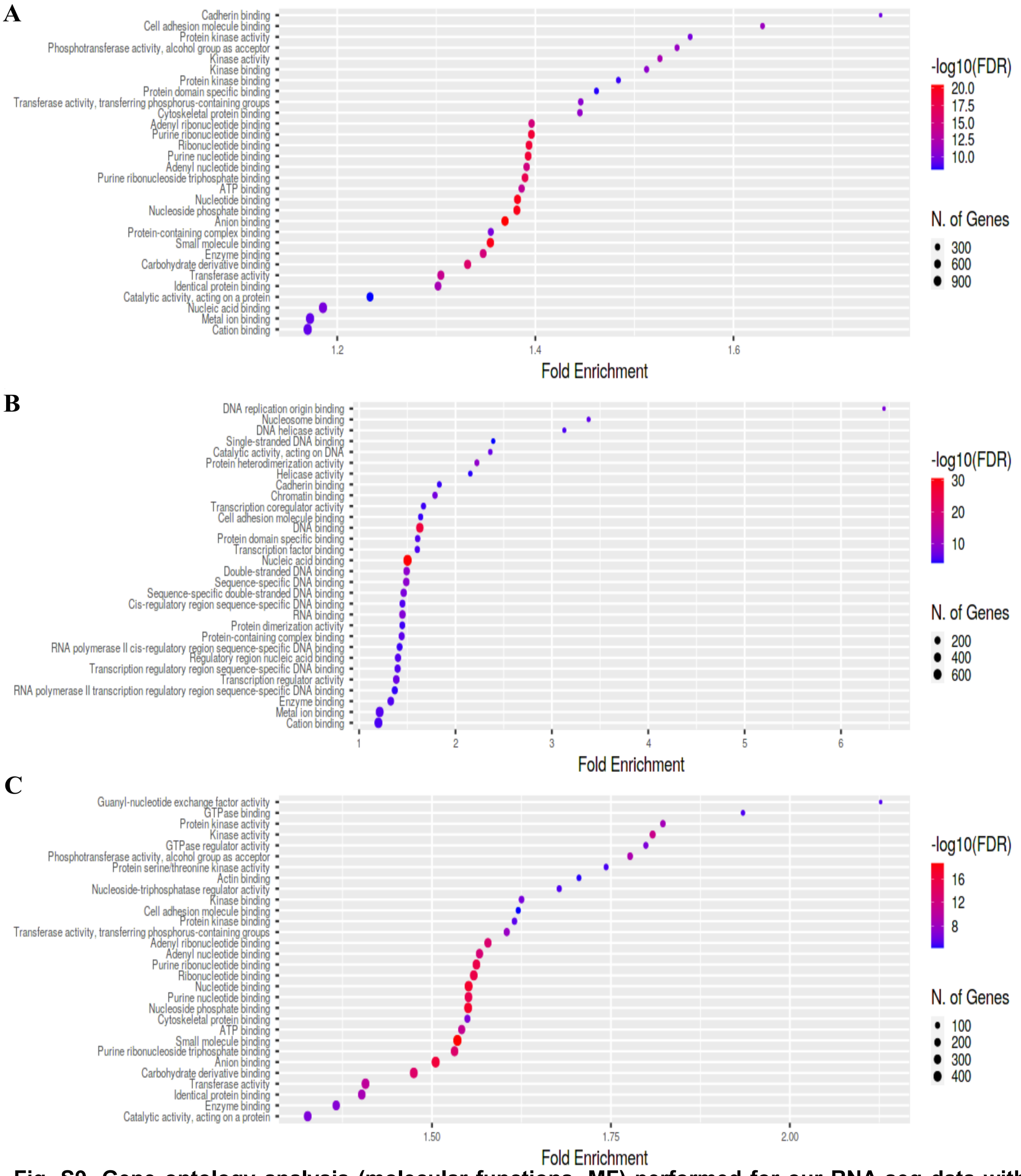

Fig. S9. Gene ontology analysis (molecular functions, MF) performed for our RNA-seq data with the ShinyGo app and the GO database. All graphs represent ontological terms most enriched in genes, expression of which was significantly altered after *CRNDE* silencing. In all the graphs, terms are sorted according to the decreasing fold enrichment values. **A:** terms enriched in genes expression of which was both increased or decreased (FC ≤ 0.8 or FC ≥ 1.2) after *CRNDE* silencing; **B:** terms enriched in genes with increased expression after *CRNDE* silencing (FC ≥ 1.2); **C:** terms enriched in genes with decreased expression after *CRNDE* silencing (FC ≤ 0.8), when compared to the control cell lines.

Table S2. Gene ontology analysis (MF) performed for our RNA-seq data with the EnrichR app and the GO database.

A

Mollecular Function FC ≤ 0.8 or FC ≥ 1.2				
#	Ontological terms	Ratio of identified genes in each category	Percentage of identified genes in each category	Adj. p-value
1.	cadherin binding (GO:0045296)	127/322	39.4%	2.36E-04
2.	DNA replication origin binding (GO:0003688)	17/23	73.9%	1.57E-03
3.	protein serine/threonine kinase activity (GO:0004674)	126/344	36.6%	7.09E-03
4.	purine ribonucleoside triphosphate binding (GO:0035639)	160/460	34.8%	1.04E-02
5.	actin binding (GO:0003779)	71/177	40.1%	1.04E-02
6.	protein kinase binding (GO:0019901)	173/506	34.2%	1.04E-02
7.	GTPase activator activity (GO:0005096)	121/336	36.0%	1.04E-02
8.	transmembrane receptor protein kinase activity (GO:0019199)	30/60	50,0%	1.05E-02
9.	transmembrane receptor protein tyrosine kinase activity (GO:0004714)	30/60	50.0%	1.05E-02
10.	kinase binding (GO:0019900)	158/461	34.3%	1.20E-02
11.	DNA binding (GO:0003677)	261/811	32.2%	1.25E-02
12.	guanyl-nucleotide exchange factor activity (GO:0005085)	60/149	40.3%	1.52E-02
13.	oxidoreductase activity, acting on the CH-CH group of donors, NAD or NADP as acceptor (GO:0016628)	12/18	66.7%	3.64E-02
14.	adenyl ribonucleotide binding (GO:0032559)	107/306	35.0%	4.67E-02

B

Molecular function FC ≥ 1.2				
#	Ontological terms	Ratio of identified genes in each category	Percentage of identified genes in each category	Adj. p-value
1.	DNA binding (GO:0003677)	181/811	22.3%	3.95E-10
2.	DNA replication origin binding (GO:0003688)	16/23	69.6%	4.22E-07
3.	RNA binding (GO:0003723)	245/1406	17.4%	8.46E-04
4.	nucleosomal DNA binding (GO:0031492)	14/33	42.4%	7.97E-03
5.	single-stranded DNA binding (GO:0003697)	28/97	28.9%	7.97E-03
6.	single-stranded DNA helicase activity (GO:0017116)	10/19	52.6%	7.97E-03
7.	3'-5' DNA helicase activity (GO:0043138)	9/16	56.3%	8.32E-03
8.	nuclear receptor coactivator activity (GO:0030374)	17/53	32.1%	4.01E-02

C

Molecular function FC ≤ 0.8				
#	Ontological terms	Ratio of identified genes in each category	Percentage of identified genes in each category	Adj. p-value
1.	transmembrane receptor protein tyrosine kinase activity (GO:0004714)	21/60	35.0%	9.98E-03
2.	GTPase activator activity (GO:0005096)	71/336	21.1%	9.98E-03
3.	guanyl-nucleotide exchange factor activity (GO:0005085)	38/149	25.5%	9.98E-03
4.	transmembrane receptor protein kinase activity (GO:0019199)	20/60	33.3%	9.98E-03
5.	GTPase binding (GO:0051020)	47/201	23.4%	9.98E-03
6.	protein serine/threonine kinase activity (GO:0004674)	70/344	20.3%	1.93E-02
7.	aminoacyl-tRNA ligase activity (GO:0004812)	14/38	36.8%	2.75E-02
8.	small GTPase binding (GO:0031267)	40/175	22.9%	3.61E-02
9.	protein kinase binding (GO:0019901)	93/506	18.4%	4.76E-02
10.	microtubule binding (GO:0008017)	49/232	21.1%	4.76E-02

Ontological terms most enriched in genes, expression of which was significantly altered after *CRNDE* silencing, are sorted according to the increasing FDR-adjusted p-value. In table **A**, terms most enriched in all differentially expressed genes (up- and downregulated) are shown; Tables **B** and **C** include terms most enriched in up-regulated genes (FC ≥ 1.2) and down-regulated genes (FC ≤ 0.8), respectively, when compared to the control cells. FC – fold change.

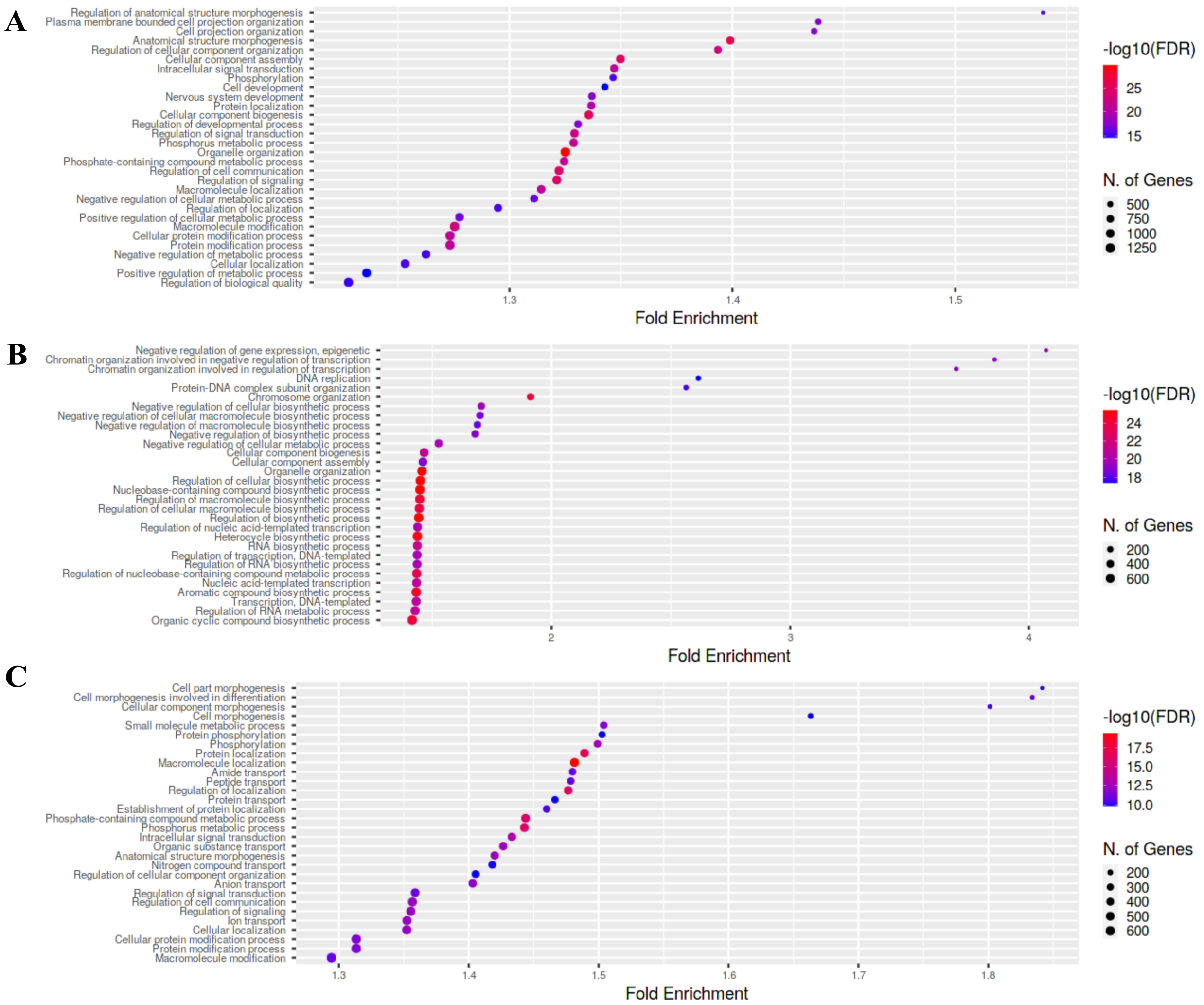

Fig. S10. Gene ontology analysis (biological processes, BP) performed for our RNA-seq data with the ShinyGo app and the GO database. All graphs represent ontological terms most enriched in genes, expression of which was significantly altered after *CRNDE* silencing. In all the graphs, terms are sorted according to the decreasing fold enrichment values. **A:** terms enriched in genes expression of which was both increased or decreased (FC ≤ 0.8 or FC ≥ 1.2) after *CRNDE* silencing; **B:** terms enriched in genes with increased expression after *CRNDE* silencing (FC ≥ 1.2); **C:** terms enriched in genes with decreased expression after *CRNDE* silencing (FC ≤ 0.8), when compared to the control cell lines.

Table S3. Gene ontology analysis (BP) performed for our RNA-seq data with the EnrichR app and the GO database.

A

#	Ontological terms	Ratio of identified genes in each category	Percentage of identified genes in each category	Adj. p-value
	**Biological process FC ≤ 0.8 or FC ≥ 1.2**			
1.	extracellular matrix organization (GO:0030198)	32/300	10.7%	3.48E-05
2.	extracellular structure organization (GO:0043062)	26/216	12.0%	3.48E-05
3.	external encapsulating structure organization (GO:0045229)	26/217	12.0%	3.48E-05
4.	supramolecular fiber organization (GO:0097435)	29/351	8.3%	1.04E-02
5.	semaphorin-plexin signaling pathway (GO:0071526)	8/35	22.9%	1.19E-02
6.	negative regulation of cell adhesion (GO:0007162)	11/73	15.1%	1.61E-02
7.	positive regulation of transcription, DNA-templated (GO:0045893)	67/1183	5.7%	1.61E-02
8.	protein localization to membrane (GO:0072657)	19/195	9.7%	1.61E-02
9.	positive regulation of vasculature development (GO:1904018)	13/102	12.7%	1.61E-02
10.	negative regulation of cell-cell adhesion (GO:0022408)	8/41	19.5%	2.01E-02

B

#	Ontological terms	Ratio of identified genes in each category	Percentage of identified genes in each category	Adj. p-value
	**Biological process FC ≥ 1.2**			
1.	DNA-dependent DNA replication (GO:0006261)	54/129	41.9%	4.10E-12
2.	double-strand break repair via homologous recombination (GO:0000724)	41/97	42.3%	2.69E-09
3.	DNA replication initiation (GO:0006270)	22/38	57.9%	1.51E-07
4.	DNA strand elongation involved in DNA replication (GO:0006271)	14/18	77.8%	9.19E-07
5.	regulation of transcription, DNA-templated (GO:0006355)	387/2244	17.2%	5.99E-06
6.	protein-DNA complex assembly (GO:0065004)	45/143	31.5%	8.05E-06
7.	chromatin assembly (GO:0031497)	29/73	39.7%	8.57E-06
8.	double-strand break repair (GO:0006302)	49/164	29.9%	8.94E-06
9.	nucleosome organization (GO:0034728)	32/94	34.0%	8.83E-05
10.	DNA repair (GO:0006281)	71/298	23.8%	1.56E-04

C

#	Ontological terms	Ratio of identified genes in each category	Percentage of identified genes in each category	Adj. p-value
	**Biological process FC ≤ 0.8**			
1.	regulation of kinase activity (GO:0043549)	31/94	33.0%	6.06E-07
2.	regulation of anatomical structure morphogenesis (GO:0022603)	36/123	29.3%	2.02E-06
3.	positive regulation of cellular catabolic process (GO:0031331)	39/141	27.7%	3.61E-06
4.	cellular response to peptide hormone stimulus (GO:0071375)	31/106	29.2%	1.03E-05
5.	cytosolic transport (GO:0016482)	33/116	28.4%	1.03E-05
6.	response to insulin (GO:0032868)	26/84	31.0%	1.73E-05
7.	protein phosphorylation (GO:0006468)	98/496	19.8%	2.05E-05
8.	regulation of actin filament-based process (GO:0032970)	23/73	31.5%	3.80E-05
9.	regulation of cytoskeleton organization (GO:0051493)	31/112	27.7%	3.41E-05
10.	response to cytokine (GO:0034097)	38/150	25.3%	4.20E-05

Ontological terms most enriched in genes, expression of which was significantly altered after *CRNDE* silencing, are sorted according to the increasing FDR-adjusted p-value. In table **A**, terms most enriched in all differentially expressed genes (up- and downregulated) are shown; Tables **B** and **C** include terms most enriched in up-regulated genes (FC ≥ 1.2) and down-regulated genes (FC ≤ 0.8), respectively, when compared to the control cells. FC – fold change.

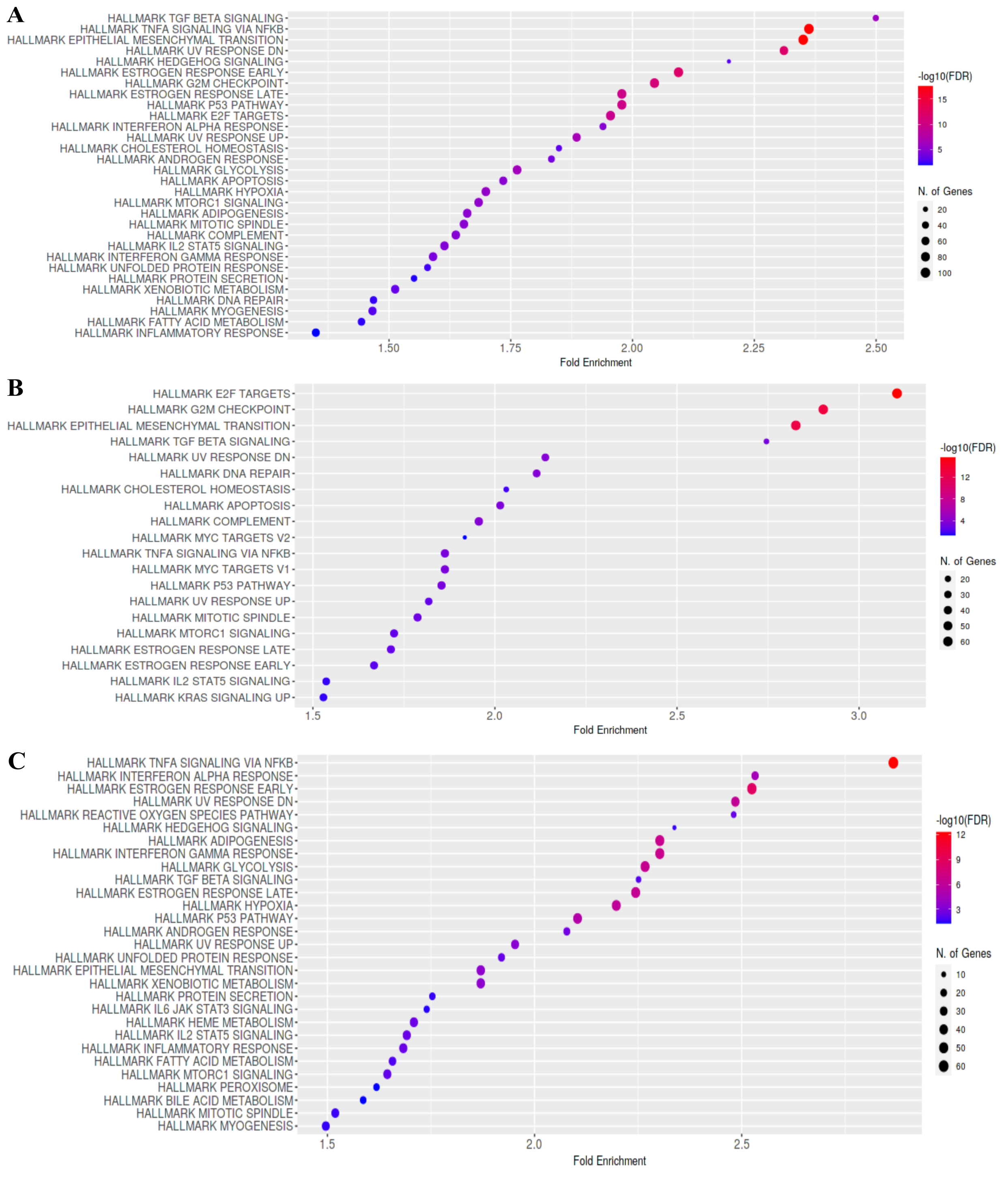

Fig. S11. Gene ontology analysis (biological processes, BP) performed for our RNA-seq data with the ShinyGo app and the MSigDB database. All graphs represent ontological terms most enriched in genes, expression of which was significantly altered after *CRNDE* silencing. In all the graphs, terms are sorted according to the decreasing fold enrichment values. **A:** terms enriched in genes expression of which was both increased or decreased (FC ≤ 0.8 or FC ≥ 1.2) after *CRNDE* silencing; **B:** terms enriched in genes with increased expression after *CRNDE* silencing (FC ≥ 1.2); **C:** terms enriched in genes with decreased expression after *CRNDE* silencing (FC ≤ 0.8), when compared to the control cell lines.

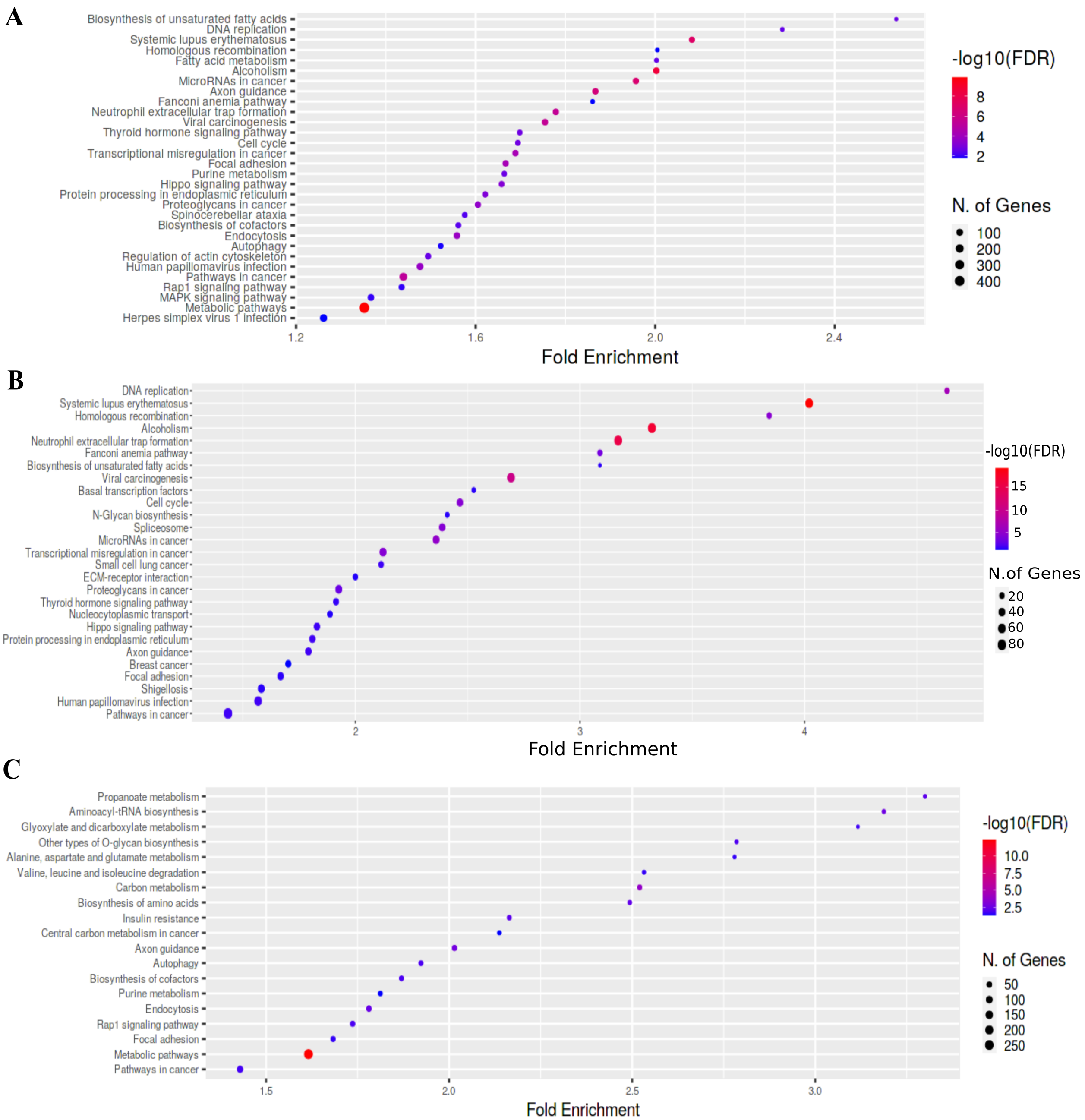

Fig. S12. Gene ontology analysis (biological processes, BP) performed for our RNA-seq data with the ShinyGo app and the KEGG database. All graphs represent ontological terms most enriched in genes, expression of which was significantly altered after *CRNDE* silencing. In all the graphs, terms are sorted according to the decreasing fold enrichment values. **A:** terms enriched in genes expression of which was both increased or decreased (FC ≤ 0.8 or FC ≥ 1.2) after *CRNDE* silencing; **B:** terms enriched in genes with increased expression after *CRNDE* silencing (FC ≥ 1.2); **C:** terms enriched in genes with decreased expression after *CRNDE* silencing (FC ≤ 0.8), when compared to the control cell lines.

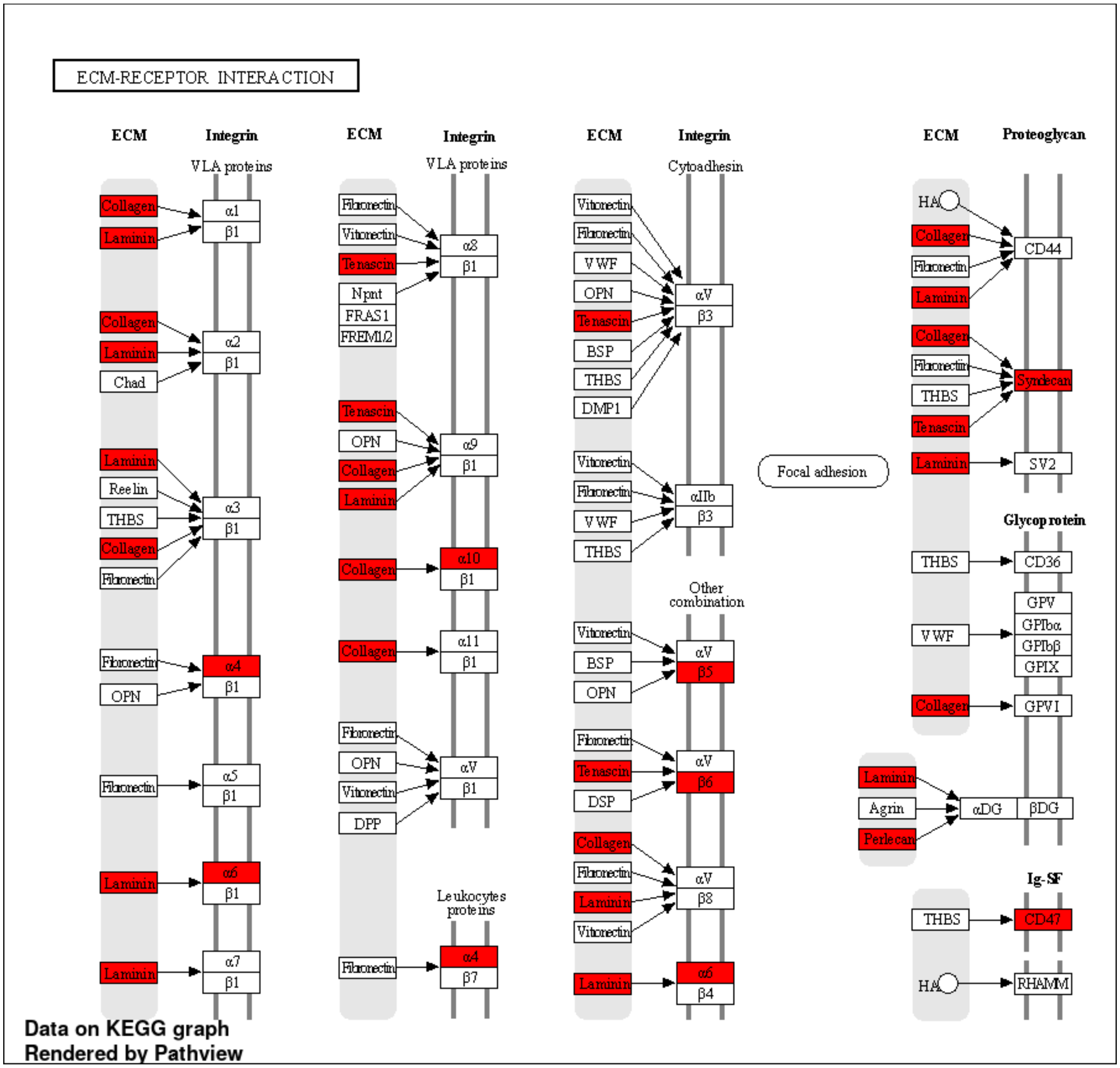

Fig. S13. Changes in the expression of genes involved in ECM-cell receptor interactions. Genes the expression of which was significantly increased in SK-OV-3 cells with *CRNDE* knockdown are marked red. In the analyzed gene set, there were no genes with significantly decreased expression in SK-OV-3 cells with the *CRNDE* knockdown compared to the control cells. The scheme was created in the ShinyGO app with the use of the KEGG database. ECM – extracellular matrix.

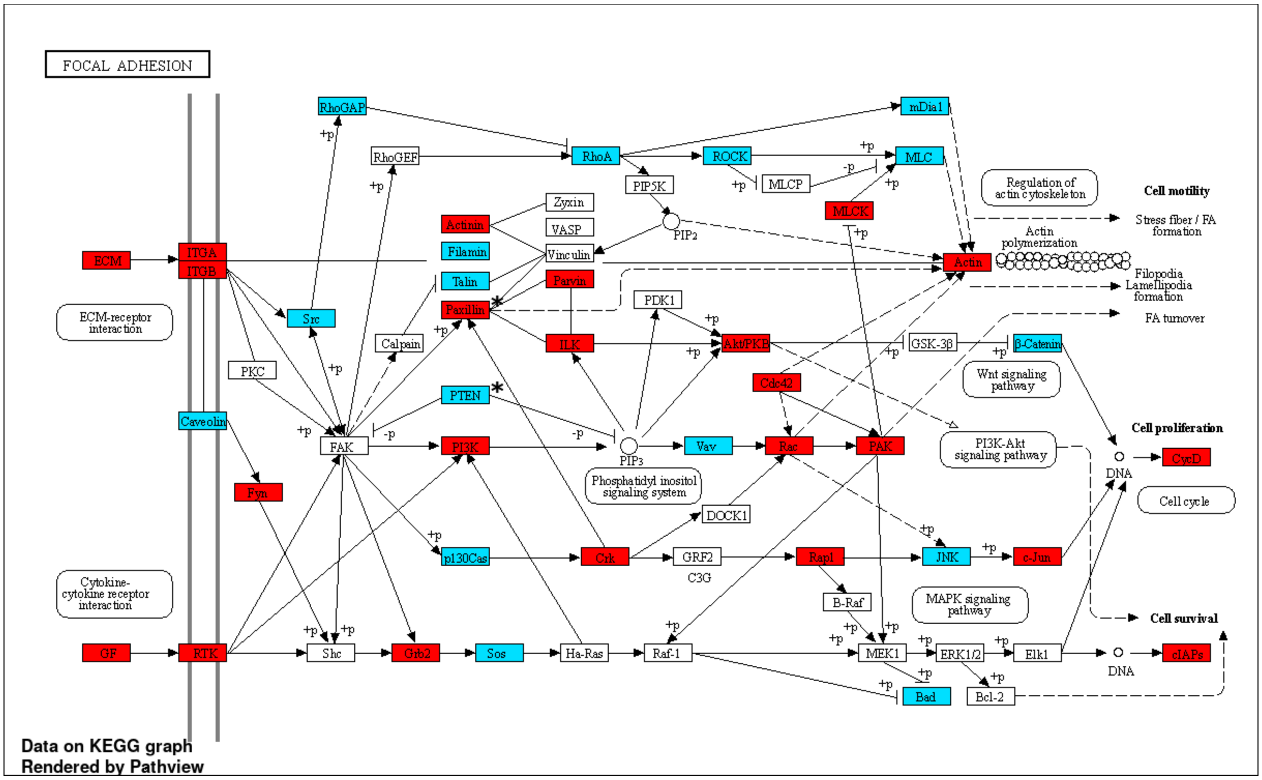

Fig. S14. Changes in the expression of genes involved in the formation of focal adhesion plaques. Genes the expression of which was significantly increased or decreased in SK-OV-3 cells with *CRNDE* knockdown are marked red or blue, respectively. The scheme was created in the ShinyGO app with the use of the KEGG database.

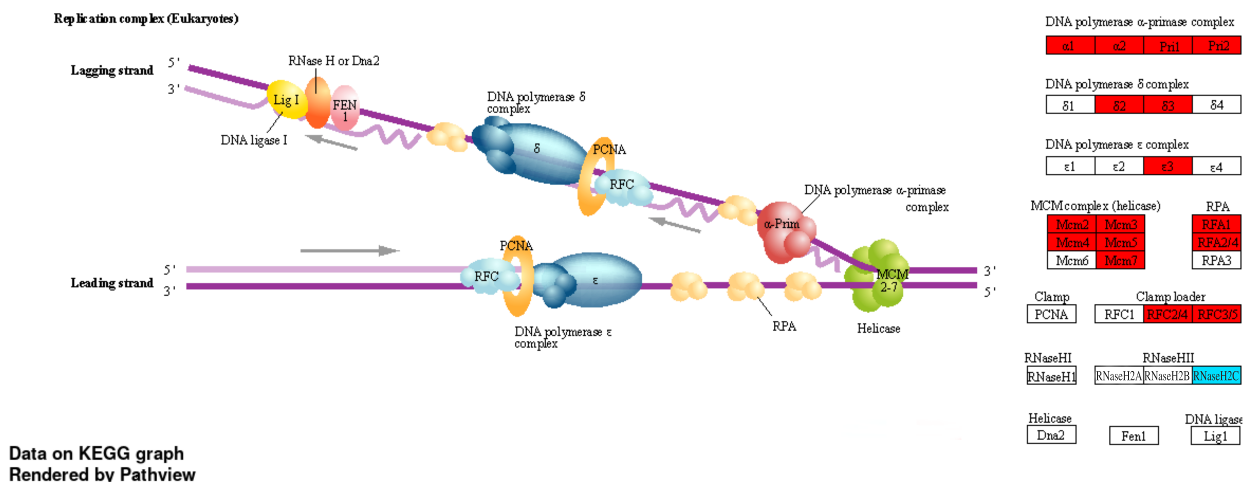

Fig. S15. Changes in the expression of genes involved in the regulation of DNA replication. Genes the expression of which was significantly increased or decreased in SK-OV-3 cells with *CRNDE* knockdown are marked red or blue, respectively. The scheme was created in the ShinyGO app with the use of the KEGG database.

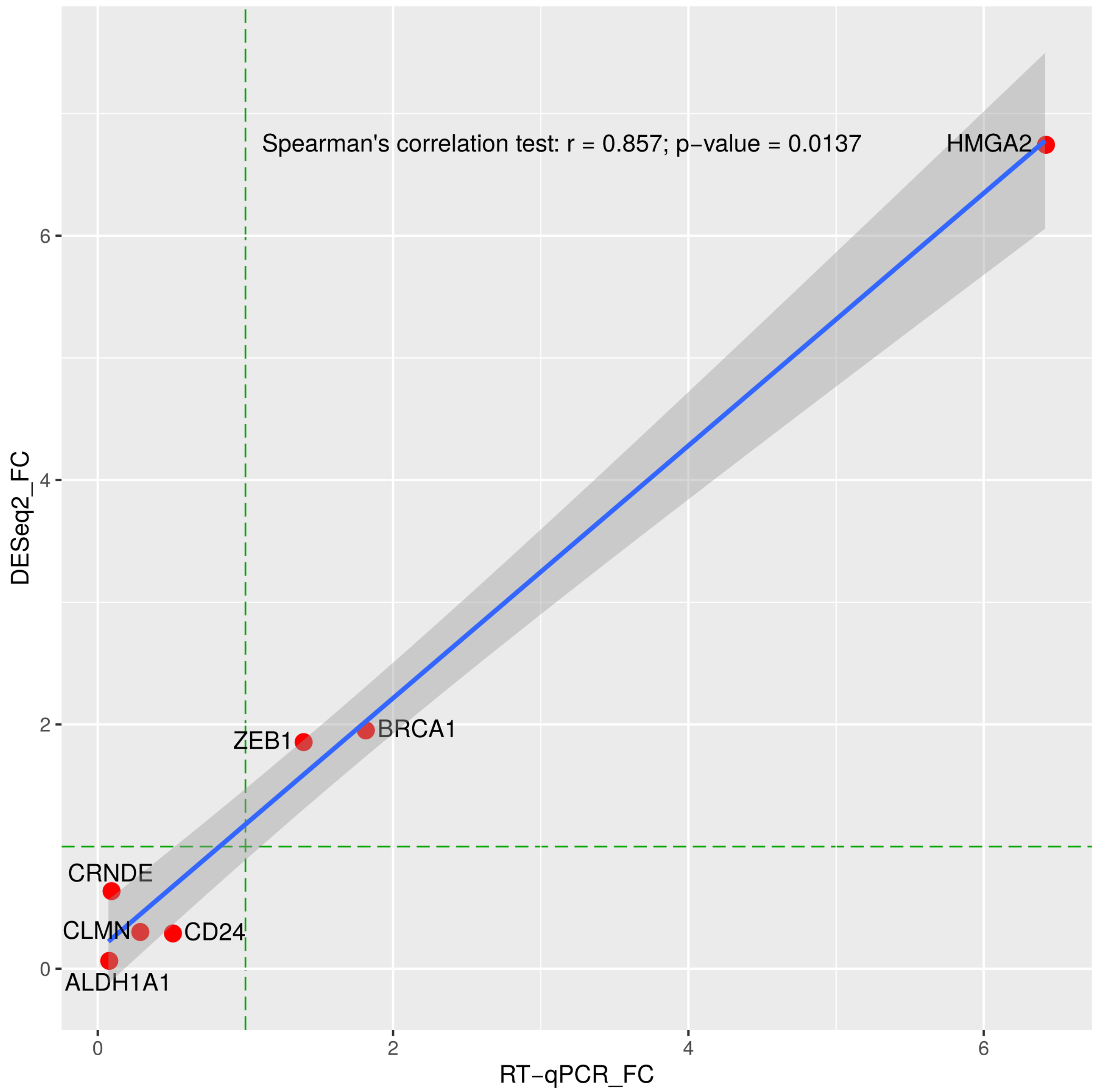

Fig. S16. Correlation of gene expression results obtained in the NGS and RT-qPCR analyses. The expression fold change (FC) values for seven genes, *CRNDE*, *CLMN*, *CD24*, *ALDH1A1*, *ZEB1*, *BRCA1* and *HMGA2* were compared for the RT-qPCR results (x-axis) and the NGS RNA-seq data analyzed with DESeq2 (y-axis). This comparison revealed a strong correlation between the outcomes of both techniques, proved with the Spearman's correlation test, thereby validating the computational algorithms used herein for the NGS analysis.

Table S4. Identification of endogenous CRNDEP in one hgOvCa sample by MS.

Sequence	Mass [Da]	Mass Δ [ppm]
VEMKNIQPLVFEISCDVFQSR + Oxidation (M)	2543.200833	-8.4
YVLGIPVYRL	1191.702165	0.5
EHGKIKVLEWFK	1512.840465	-3.2
YVLGIPVYR	1078.621218	3.4
IKVLEWFKYVLGIPVYR	2122.239396	2.4
EDKEDATGNVEMK	1464.633076	-4.9
**Full sequence coverage**	MLAEIHPKAG LQS***LQFIMEL*** ***LYWLLEGGDS***<br>***EDKEDATGNV EMKNIQPLVF EISCDVFQSR***<br>***CKEHGKIKVL EWFKYVLGIP VYRL***	

CRNDEP amino acids identified herein by MS are denoted with green letters. Those identified in the study by Chothani et al. [6] based on Ribo-seq data are written in bold and italics, while the oligopeptide confirmed in their MS/MS analysis is underlined. The region matching a synthetic epitope that we used to develop the anti-CRNDEP antibody is highlighted in yellow; hgOvCa – high-grade ovarian carcinoma.

Table S5. A joined list of CRNDEP protein partners identified by mass spectrometry.

#	Protein ID	Protein name	Gene name	CRNDEP fused with Strep-Flag		CRNDEP (endogenous)	
				Peptides	Coverage	Peptides	Coverage
1	O43663	Protein regulator of cytokinesis 1	*PRC1*	14	25.32	14	28.87
2	Q5SW79	Centrosomal protein of 170 kDa	*CEP170*	13	12.75	11	9.85
3	P01024	Complement C3	*C3*	9	5.05	13	7.16
4	Q96SI1	BTB/POZ domain-containing protein KCTD15	*KCTD15*	8	31.8	13	51.94
5	P26196	Probable ATP-dependent RNA helicase DDX6	*DDX6*	7	25.26	7	28.78
6	Q15717	ELAV-like protein 1	*ELAVL1*	4	14.42	6	22.39
7	P0C0L4	Complement C4-A,Complement C4-B	*C4A,C4B*	3	2.24	6	4.01
8	Q9NZB2	Constitutive coactivator of PPAR-gamma-like protein 1	*FAM120A*	3	3.76	6	7.96
9	P16989	Y-box-binding protein 3	*YBX3*	2	8.06	4	23.12
10	O00425	Insulin-like growth factor 2 mRNA-binding protein 3	*IGF2BP3*	2	3.97	4	8.98
11	Q12905	Interleukin enhancer-binding factor 2	*ILF2*	3	10.51	3	10.51
12	O00410	Importin-5	*IPO5*	3	3.28	2	3.01
13	Q719H9	BTB/POZ domain-containing protein KCTD1	*KCTD1*	2	8.56	3	8.56
14	Q12830	Nucleosome-remodeling factor subunit BPTF	*BPTF*	2	1.12	2	0.66
15	**B8Y2T7**	**HCG1815491 (CRNDEP)**	***CRNDE***	**4**	**27.38**		
16	P61978	Heterogeneous nuclear ribonucleoprotein K	*HNRNPK*	13	43.2		
17	P52209	6-phosphogluconate dehydrogenase, decarboxylating	*PGD*	4	12.22		
18	P11586	C-1-tetrahydrofolate synthase, cytoplasmic	*MTHFD1*	5	9.3		
19	Q99729	Heterogeneous nuclear ribonucleoprotein A/B	*HNRNPAB*	6	21.39		
20	P01889	HLA class I histocompatibility antigen	*HLA-B*	2	7.46		
21	P09914	Interferon-induced protein with tetratricopeptide repeats 1	*IFIT1*	3	9.83		
22	P35221	Catenin alpha-1	*CTNNA1*	3	7.95		
23	Q9C005	Protein dpy-30 homolog	*DPY30*	2	36.36		
24	P08865	40S ribosomal protein SA	*RPSA*	2	13.56		
25	Q13813	Spectrin alpha chain, non-erythrocytic 1	*SPTAN1*	3	2.1		
26	P23528	Cofilin-1	*CFL1*	2	16.87		
27	Q9Y281	Cofilin-2	*CFL2*	2	16.87		
28	P55072	Transitional endoplasmic reticulum ATPase	*VCP*	7	13.52		
29	Q01082	Spectrin beta chain, non-erythrocytic 1	*SPTBN1*	2	1.06		
30	Q08J23	tRNA (cytosine(34)-C(5))-methyltransferase	*NSUN2*	3	7.43		
31	P31150	Rab GDP dissociation inhibitor alpha	*GDI1*	2	7.16		
32	P27824	Calnexin	*CANX*	2	5.74		
33	P33240	Cleavage stimulation factor subunit 2	*CSTF2*	3	11.09		
34	Q8TEX9	Importin-4	*IPO4*	2	2.04		
35	P78371	T-complex protein 1 subunit beta	*CCT2*	2	4.49		
36	P27695	DNA-(apurinic or apyrimidinic site) lyase	*APEX1*	2	8.49		
37	P30153	Serine/threonine-protein phosphatase 2A 65 kDa regulatory subunit A alpha isoform	*PPP2R1A*	4	10.7		
38	P61604	10 kDa heat shock protein, mitochondrial	*HSPE1*	2	21.57		
39	Q92616	eIF-2-alpha kinase activator GCN1	*GCN1*	2	1.2		
40	Q01813	ATP-dependent 6-phosphofructokinase, platelet type	*PFKP*	2	2.81		
41	P49368	T-complex protein 1 subunit gamma	*CCT3*	3	11.01		
42	P22392	Nucleoside diphosphate kinase B	*NME2*	2	21.05		
43	O00148,Q13838	ATP-dependent RNA helicase DDX39A,Spliceosome RNA helicase DDX39B	*DDX39A*	2	4.68		
44	P14324	Farnesyl pyrophosphate synthase	*FDPS*	2	7.16		
45	P60174	Triosephosphate isomerase	*TPI1*	3	19.93		
46	P15531	Nucleoside diphosphate kinase A	*NME1*	2	17.76		
47	P35232	Prohibitin	*PHB*	4	20.96		
48	Q9Y3I0	tRNA-splicing ligase RtcB homolog	*RTCB*	3	7.52		
49	Q07065	Cytoskeleton-associated protein 4	*CKAP4*	3	6.48		
50	Q92499	ATP-dependent RNA helicase DDX1	*DDX1*	2	5.0		
51	P17987	T-complex protein 1 subunit alpha	*TCP1*	2	3.78		
52	P00403	Cytochrome c oxidase subunit 2	*MT-CO2*	2	13.22		
53	Q9Y6C9	Mitochondrial carrier homolog 2	*MTCH2*	2	7.26		
54	P30041	Peroxiredoxin-6	*PRDX6*	2	8.93		
55	P31930	Cytochrome b-c1 complex subunit 1, mitochondrial	*UQCRC1*	2	6.46		
56	Q7L2E3	Putative ATP-dependent RNA helicase DHX30	*DHX30*	2	2.93		
57	P14618	Pyruvate kinase PKM	*PKM*	6	15.25		
58	Q09028	Histone-binding protein RBBP4	*RBBP4*	2	8.0		
59	P02786	Transferrin receptor protein 1	*TFRC*	3	5.26		
60	P22234	Multifunctional protein ADE2	*PAICS*	4	11.29		
61	P08195	4F2 cell-surface antigen heavy chain	*SLC3A2*	5	9.68		
62	P51648	Fatty aldehyde dehydrogenase	*ALDH3A2*	2	4.54		

63	Q13501	Sequestosome-1	*SQSTM1*	3	10.23		
64	P36871	Phosphoglucomutase-1	*PGM1*	2	4.09		
65	Q8WWM7	Ataxin-2-like protein	*ATXN2L*	2	2.42		
66	Q9NR12	PDZ and LIM domain protein 7	*PDLIM7*	5	14.0		
67	P19474	E3 ubiquitin-protein ligase TRIM21	*TRIM21*	2	5.89		
68	Q13263	Transcription intermediary factor 1-beta	*TRIM28*	4	5.03		
69	P30101	Protein disulfide-isomerase A3	*PDIA3*	4	11.88		
70	P41250	Glycine--tRNA ligase	*GARS*	2	2.44		
71	P12268	Inosine-5'-monophosphate dehydrogenase 2	*IMPDH2*	2	5.84		
72	P56134	ATP synthase subunit f, mitochondrial	*ATP5J2*	2	25.53		
73	Q9UN86	Ras GTPase-activating protein-binding protein 2	*G3BP2*	2	5.19		
74	Q9NZM1	Myoferlin	*MYOF*	2	0.97		
75	P12277	Creatine kinase B-type	*CKB*	4	20.21		
76	Q9NX20	39S ribosomal protein L16, mitochondrial	*MRPL16*	2	10.36		
77	P31939	Bifunctional purine biosynthesis protein PURH	*ATIC*	2	4.39		
78	P14868	Aspartate--tRNA ligase, cytoplasmic	*DARS*	2	3.99		
79	Q14204	Cytoplasmic dynein 1 heavy chain 1	*DYNC1H1*	5	1.68		
80	Q7Z417	Nuclear fragile X mental retardation-interacting protein 2	*NUFIP2*	2	3.17		
81	Q96HC4	PDZ and LIM domain protein 5	*PDLIM5*	2	4.03		
82	O95373	Importin-7	*IPO7*	2	2.99		
83	O14980	Exportin-1	*XPO1*	3	4.67		
84	P62633	Cellular nucleic acid-binding protein	*CNBP*	2	10.17		
85	P19623	Spermidine synthase	*SRM*	2	11.26		
86	P30040	Endoplasmic reticulum resident protein 29	*ERP29*	2	13.79		
87	P07814	Bifunctional glutamate/proline--tRNA ligase	*EPRS*	4	4.03		
88	P14923	Junction plakoglobin	*JUP*			2	3.89
89	P07910	Heterogeneous nuclear ribonucleoproteins C1/C2	*HNRNPC*			9	28.1
90	Q96PK6	RNA-binding protein 14	*RBM14*			10	18.24
91	P42167,P42166	Lamina-associated polypeptide 2, isoforms beta/gamma, Lamina-associated polypeptide 2, isoform alpha	*TMPO*			3	12.78
92	P50993,P13637	Sodium/potassium-transporting ATPase subunit alpha-2,Sodium/potassium-transporting ATPase subunit alpha-3	*ATP1A2,ATP1A3*			2	3.04
93	P48643	T-complex protein 1 subunit epsilon	*CCT5*			3	9.24
94	Q13435	Splicing factor 3B subunit 2	*SF3B2*			3	3.91
95	P27797	Calreticulin	*CALR*			2	14.39
96	P22087	rRNA 2'-O-methyltransferase fibrillarin	*FBL*			2	11.84
97	Q8WUM4	Programmed cell death 6-interacting protein	*PDCD6IP*			2	4.95
98	Q96E39	RNA binding motif protein, X-linked-like-1	*RBMXL1*			2	6.92
99	P62888	60S ribosomal protein L30	*RPL30*			3	29.57
100	Q14847	LIM and SH3 domain protein 1	*LASP1*			2	10.34
101	Q9NZI8	Insulin-like growth factor 2 mRNA-binding protein 1	*IGF2BP1*			3	6.93
102	P19338	Nucleolin	*NCL*			3	4.65
103	Q9Y597	BTB/POZ domain-containing protein KCTD3	*KCTD3*			2	5.03
104	Q14CN4	Keratin, type II cytoskeletal 72	*KRT72*			2	2.35
105	O00231	26S proteasome non-ATPase regulatory subunit 11	*PSMD11*			2	5.69
106	P07358	Complement component C8 beta chain	*C8B*			2	5.08
107	P61088	Ubiquitin-conjugating enzyme E2 N	*UBE2N*			2	20.39
108	Q9UKM9	RNA-binding protein Raly	*RALY*			5	18.95
109	Q08554	Desmocollin-1	*DSC1*			3	5.37
110	Q6VMQ6	Activating transcription factor 7-interacting protein 1	*ATF7IP*			2	3.54
111	Q6P1L8	39S ribosomal protein L14, mitochondrial	*MRPL14*			3	13.79
112	P13804	Electron transfer flavoprotein subunit alpha, mitochondrial	*ETFA*			2	10.21
113	P0DI83	Ras-related protein Rab-34, isoform NARR	*RAB34*			2	18.18
114	P45974	Ubiquitin carboxyl-terminal hydrolase 5	*USP5*			2	3.61
115	O95613	Pericentrin	*PCNT*			4	1.47
116	P53992	Protein transport protein Sec24C	*SEC24C*			2	3.11
117	Q08043	Alpha-actinin-3	*ACTN3*			2	2.89
118	O75367	Core histone macro-H2A.1	*H2AFY*			2	8.33
119	P54136	Arginine--tRNA ligase, cytoplasmic	*RARS*			2	3.48
120	P54886	Delta-1-pyrroline-5-carboxylate synthase	*ALDH18A1*			2	2.52
121	P60953	Cell division control protein 42 homolog	*CDC42*			2	14.66
122	Q4VC31	Coiled-coil domain-containing protein 58	*CCDC58*			2	18.06

Proteins detected as interactants of both the CRNDEP-Strep-Flag fusion protein and the endogenous CRNDEP micropeptide are highlighted in yellow. The CRNDEP micropeptide is marked red.

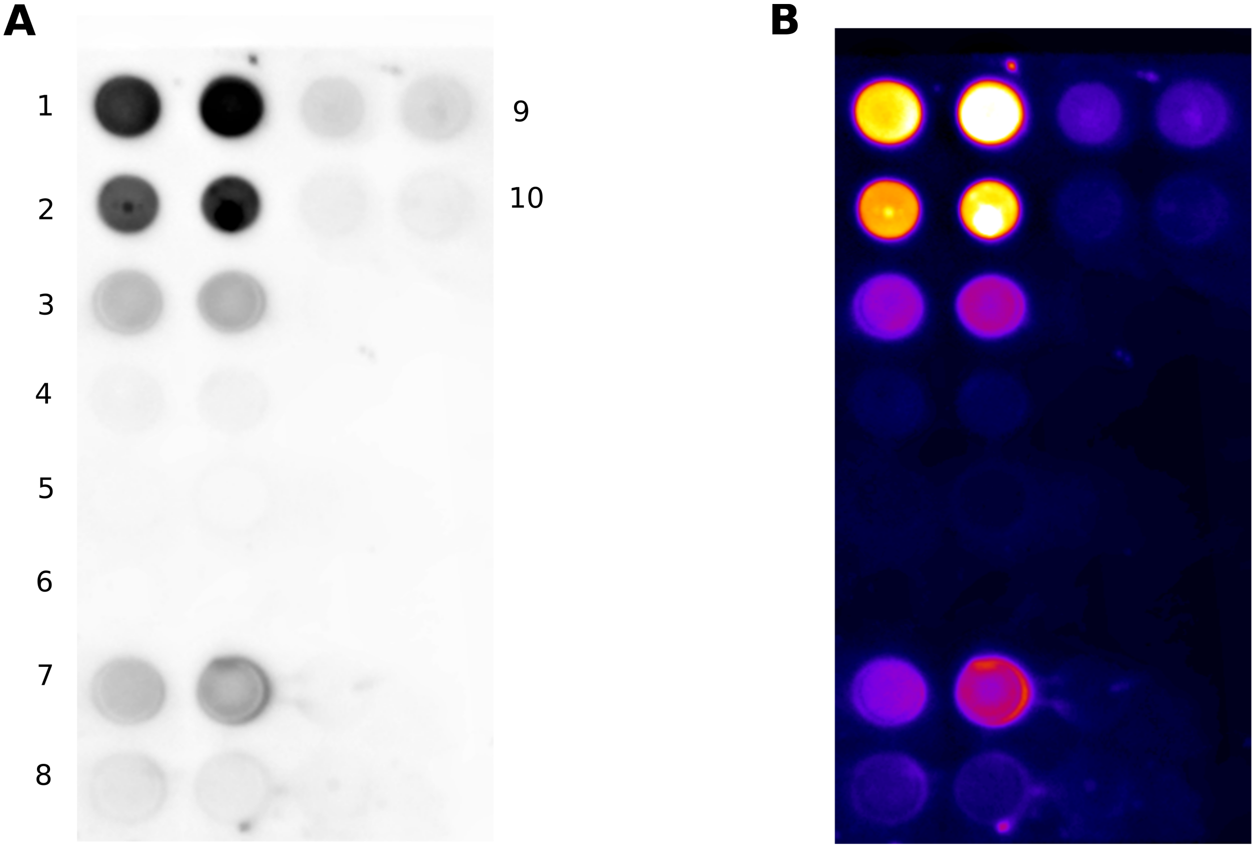

Fig. S17. Dot-blot analysis of CRNDEP sensitivity to different physico-chemical factors. 10 μg of the total protein lysate obtained from a single hgOvCa sample were added to each well. The experiment was performed on a nitrocelulose membrane. A: original dot blot result, B: a heatmap representation of the dot blot, generated by the ImageJ app. 1: lysate + H_2O (ctrl); 2: lysate + H_2O heated to 100 ºC (5 min); 3: lysate + 10mM DTT; 4: lysate + 10mM DTT heated to 100 ºC (5 min); 5: lysate + 1x Laemmli buffer; 6: lysate + 1x Laemmli buffer heated to 100 ºC (5 min); 7: lysate + 1% SDS; 8: lysate + 1% SDS heated to 100 ºC (5 min); 9: lysate + 2% SDS; 10: lysate + 2% SDS heated to 100 ºC (5 min). Each experimental variant was carried out in two horizontally oriented technical replications.

Table S6. Ontology analysis (cellular components, CC) for CRNDEP protein interactants found herein.

#	Cellular component - Ontological terms	Ratio of identified proteins in each category	p-value	Adjusted p-value	Identified proteins
1	cytoplasmic stress granule (GO:0010494)	5/41	8.2E-07	1.071E-04	ATXN2L; PABPC4; DDX6; NUFIP2; DDX1
2	cytoplasmic ribonucleoprotein granule (GO:0036464)	7/170	8.0E-06	5.246E-04	ATXN2L; DDX6; NUFIP2; PABPC4; DDX1; SQSTM1; CKAP4
3	chaperonin-containing T-complex (GO:0005832)	3/13	2.1E-05	8.972E-04	CCT3; CCT2; TCP1
4	secretory granule lumen (GO:0034774)	8/317	6.1E-05	1.993E-03	CCT2; DYNC1H1; VCP; PKM; IMPDH2; SPTAN1; ILF2; PRDX6
5	focal adhesion (GO:0005925)	8/356	1.4E-04	3.565E-03	YWHAE; PDIA3; HNRNPK; CFL1; CTNNA1; EZR; YWHAG; PDLIM7
6	ficolin-1-rich granule lumen (GO:1904813)	5/123	1.8E-04	3.920E-03	VCP ;PKM; IMPDH2; ILF2; PGM1
7	microtubule cytoskeleton (GO:0015630)	8/388	2.4E-04	4.403E-03	CCT3; CCT2; DYNC1H1; PPP2R1A; PRC1; TCP1; SPTAN1; TUBA4A
8	microtubule (GO:0005874)	6/210	2.7E-04	4.403E-03	CCT3; CCT2; DYNC1H1; PRC1; TCP1; TUBA4A
9	polymeric cytoskeletal fiber (GO:0099513)	6/221	3.6E-04	4.403E-03	CCT3; CCT2; DYNC1H1; TCP1; EZR; TUBA4A
10	cytoskeleton (GO:0005856)	9/520	3.6E-04	4.403E-03	CCT3; PPP2R1A; EZR; PDLIM5; SPTAN1; PGM1; TUBA4A; PDLIM7; SPTBN1
11	NURF complex (GO:0016589)	2/7	3.7E-04	4.403E-03	RBBP4; BPTF
12	azurophil granule (GO:0042582)	5/154	5.1E-04	5.533E-03	CCT2; DYNC1H1; VCP; PRDX6; CKAP4
13	azurophil granule lumen (GO:0035578)	4/90	5.9E-04	5.924E-03	CCT2; DYNC1H1; VCP; PRDX6
14	ISWI-type complex (GO:0031010)	2/11	9.6E-04	8.961E-03	RBBP4; BPTF
15	ficolin-1-rich granule (GO:0101002)	5/184	1.1E-03	9.312E-03	VCP; PKM; IMPDH2; ILF2; PGM1
16	histone methyltransferase complex (GO:0035097)	3/49	1.2E-03	9.312E-03	RBBP4; DPY30; BPTF
17	nuclear body (GO:0016604)	9/618	1.2E-03	9.312E-03	DDX17 ;ATXN2L; DDX39A; NUFIP2; DDX1; APEX1; CSTF2; SQSTM1; CKAP4
18	mitochondrion (GO:0005739)	12/1026	1.3E-03	9.312E-03	DHX30; MTCH2; PKM; PPP2R1A; APEX1; UQCRC1; MT-CO2; GARS; PHB; HSPE1; PRDX6; ATP5J2
19	Set1C/COMPASS complex (GO:0048188)	2/13	1.4E-03	9.312E-03	DPY30; BPTF
20	tertiary granule lumen (GO:1904724)	3/55	1.7E-03	1.085E-02	SPTAN1; ILF2; PGM1
21	nucleolus (GO:0005730)	9/676	2.3E-03	1.422E-02	DDX17; DDX6; XPO1; DDX39A; NSUN2; DDX1; APEX1; ILF2; SPTBN1
22	cytosolic part (GO:0044445)	4/159	4.7E-03	2.697E-02	CCT3; CCT2; TCP1; RPSA
23	ribonucleoprotein granule (GO:0035770)	3/80	4.8E-03	2.697E-02	DDX6; DHX30; CKAP4
24	vacuolar lumen (GO:0005775)	4/161	4.9E-03	2.697E-02	CCT2; DYNC1H1; VCP; PRDX6
25	integral component of lumenal side of endoplasmic reticulum membrane (GO:0071556)	2/29	6.7E-03	3.525E-02	CANX; HLA-B
26	nuclear speck (GO:0016607)	5/296	8.6E-03	4.331E-02	DDX17; ATXN2L; DDX39A; APEX1; CKAP4

The analysis was performed using the EnrichR app and the GO database. Each hit is additionally supplemented with proteins enriching the given ontological term.

Table S7. Ontology analysis (molecular functions, MF) for CRNDEP protein interactants found herein.

#	Molecular function - Ontological terms	Ratio of identified proteins in each category	p-value	Adjusted p-value	Identified proteins
1	RNA binding (GO:0003723)	44/1387	1.0E-28	1.19E-25	YWHAE; DDX6; VCP; TFRC; NUFIP2; DDX1; RTCB; CSTF2; MRPL16; SLCA2; EPRS; IFIT1; ELAVL1; IPO5; SYNCRIP; DHX30; TRIM28; FAM120A; CTNNA1; IGF2BP3; YWHAG; SPTBN1; PDIA3; DDX17; CCT3; DYNC1H1; FDPS; DARS; NSUN2; PABPC4; CNBP; RPSA; HSPE1; ILF2; HNRNPAB; CKAP4; ATXN2L; PKM; DDX39A; HNRNPK; APEX1; TCP1; CANX; EZR
2	cadherin binding (GO:0045296)	15/313	4.0E-12	2.29E-09	YWHAE; DDX6; SLC3A2; PAICS; PRDX6; ATXN2L; ATIC; PKM; HNRNPK; CTNNA1; EZR; PDLIM5; SPTAN1; SPTBN1; PFKP
3	ubiquitin protein ligase binding (GO:0031625)	10/284	3.5E-07	1.34E-04	YWHAE; CCT2; VCP; TPI1; TRIM28; TCP1; UQCRC1; CKB; SQSTM1; PRDX6
4	ubiquitin-like protein ligase binding (GO:0044389)	10/297	5.3E-07	1.51E-04	YWHAE; CCT2; VCP; TPI1; TRIM28; TCP1; UQCRC1; CKB; SQSTM1; PRDX6
5	double-stranded RNA binding (GO:0003725)	5/59	5.1E-06	1.18E-03	DHX30; TFRC; SLC3A2; ELAVL1; ILF2
6	ATP dependent RNA helicase (GO:0004004)	5/67	9.7E-06	1.59E-03	DDX17; DDX6; DHX30; DDX39A; DDX1
7	helicase activity (GO:0004386)	3/34	4.0E-04	4.64E-02	DDX17; DDX6; DDX1
8	aminoacyl-tRNA ligase activity (GO:0004812)	3/41	7.0E-04	6.22E-02	DARS; GARS; EPRS
9	vinculin binding (GO:0017166)	2/10	7.9E-04	6.46E-02	RTCB; CTNNA1
10	ATPase activity, coupled (GO:0042623)	4/94	6.9E-04	6.64E-02	DDX17; RBBP4; DDX1; BPTF
11	protein kinase C binding (GO:0005080)	3/40	6.5E-04	6.84E-02	PDLIM5; SQSTM1; YWHAG
12	poly(A) binding (GO:0008143)	2/14	1.6E-03	1.21E-01	DDX1; PABPC4
13	MHC protein complex binding (GO:0023023)	2/19	2.9E-03	1.97E-01	YWHAE; PKM
14	poly-purine tract binding (GO:0070717)	2/20	3.2E-03	2.06E-01	DDX1; PABPC4
15	protein transporter activity (GO:0008565)	3/80	4.8E-03	2.91E-01	IPO7; IPO4; IPO5
16	protein kinase binding (GO:0019901)	7/495	5.1E-03	2.94E-01	PRC1; EZR; PDLIM5; ELAVL1; SQSTM1; YWHAG; TUBA4A
17	histone deacetylase binding (GO:0042826)	3/85	5.7E-03	3.12E-01	YWHAE; RBBP4; PHB
18	nuclear localization sequence binding (GO:0008139)	2/28	6.3E-03	3.29E-01	IPO4; IPO5
19	protein homodimerization activity (GO:0042803)	8/664	7.2E-03	3.46E-01	ATIC; TFRC; ERP29; DPY30; EPRS; ELAVL1; PRDX6; SRM
20	mRNA binding (GO:0003729)	4/179	7,2E-03	3,58E-01	CSTF2; IGF2BP3; ELAVL1; HNRNPAB
21	phosphoric diester hydrolase activity (GO:0008081)	2/33	8.7E-03	3.98E-01	PDIA3; APEX1
22	ATPase activity (GO:0016887)	4/203	1.1E-02	4.87E-01	DYNC1H1; VCP; DDX39A; ATP5J2

The analysis was performed using the EnrichR app and the GO database. Each hit is additionally supplemented with proteins enriching the given ontological term.

Table S8. Ontology analysis (biological processes, BP) for CRNDEP protein interactants found herein.

#	Biological process - Ontological terms	Ratio of identified proteins in each category	p-value	Adjusted p-value	Identified proteins
1	positive regulation of establishment of protein localization to telomere (GO:1904851)	3/10	2.11E-08	7.14E-06	CCT3; CCT2; TCP1
2	regulation of establishment of protein localization to telomere (GO:0070203)	3/11	2.63E-08	7.14E-06	CCT3; CCT2; TCP1
3	regulation of protein localization to Cajal body (GO:1904869)	3/11	2.92E-08	7.14E-06	CCT3; CCT2; TCP1
4	mRNA splicing via spliceosome (GO:0000398)	6/274	2.94E-06	5.33E-04	DDX39A; HNRNPK; DDX39B; DDX1; CSTF2; ELAVL1
5	purine nucleoside monophosphate biosynthetic process (GO:0009127)	3/12	9.99E-06	1.38E-03	ATIC; IMPDH2; PAICS
6	regulation of cytoplasmic translation (GO:2000765)	3/15	1.37E-05	1.38E-03	PKM; CNBP; YBX3
7	positive regulation of telomerase RNA localization to Cajal body (GO:1904874)	3/15	1.37E-05	1.38E-03	CCT3; CCT2; TCP1
8	purine ribonucleoside monophosphate biosynthetic process (GO:0009168)	3/16	1.82E-05	1.50E-03	ATIC; IMPDH2; PAICS
9	stress granule assembly (GO:0034063)	3/21	3.73E-05	2.25E-03	ATXN2L; DDX6; DYNC1H1
10	RNA splicing via transesterification reactions with bulged adenosine as nucleophile (GO:0000377)	5/251	3.73E-05	2.25E-03	DDX39A; HNRNPK; DDX39B; CSTF2; ELAVL1
11	canonical glycolysis (GO:0061621)	3/24	4.57E-05	2.44E-03	TPI1; PKM; PFKP
12	tRNA processing (GO:0008033)	4/64	7.84E-05	3.73E-03	NSUN2; DDX1; RTCB; CSTF2
13	cellular response to indole-3-methanol (GO:0071681)	2/5	9.19E-05	4.16E-03	JUP; CTNNA1
14	scaRNA localization to Cajal body (GO:0090666)	2/5	1.61E-04	6.06E-03	CCT2; TCP1
15	regulation of apoptotic cell clearance (GO:2000425)	2/8	1.94E-04	6.06E-03	C3; C4A
16	positive regulation of protein import into nucleus (GO:0042307)	3/28	1.83E-04	6.06E-03	TRIM28; JUP; IPO5
17	glycolytic process (GO:0006096)	3/29	1.90E-04	6.06E-03	TPI1; PKM; PGM1
18	positive regulation of protein import (GO:1904591)	3/30	1.94E-04	6.06E-03	TRIM28; JUP; IPO5
19	antigen processing and presentation of peptide antigen via MHC class I (GO:0002474)	3/33	1.94E-04	6.06E-03	PDIA3; CANX; HLA-B
20	positive regulation of telomere maintenance via telomerase (GO:0032212)	3/33	1.94E-04	6.06E-03	CCT3; CCT2; TCP1
21	regulation of protein import into nucleus (GO:0042306)	3/36	2.86E-04	8.36E-03	TRIM28; JUP; IPO5
22	protein import (GO:0017038)	4/89	4.22E-04	1.12E-02	ALDH3A2; IPO7; IPO4; IPO5
23	positive regulation of transcription DNA-templated (GO:0045893)	11/1183	4.22E-04	1.12E-02	TRIM28; DDX39B; JUP; CNBP; APEX1; NME2; PHB; ILF2; SQSTM1; HNRNPAB; BPTF
24	DNA replication-dependent nucleosome assembly (GO:0006335)	2/10	5.39E-04	1.30E-02	RBBP4; IPO4
25	carbohydrate catabolic process (GO:0016052)	3/41	5.39E-04	1.30E-02	TPI1; PKM; PGM1
26	positive regulation of translation (GO:0045727)	4/100	5.47E-04	1.30E-02	PKM; CNBP; ELAVL1; YBX3

The analysis was performed using the EnrichR app and the GO database. Each hit is additionally supplemented with proteins enriching the given ontological term.

Table S9. Ontology analysis for CRNDEP protein interactants found herein performed in the context of biological pathways.

	Biological pathways - Ontological terms	Ratio of identified proteins in each category	Percentage of identified proteins in each category	p – value	Adj. p - value	Identified proteins
1	Assembly of the primary cilium Homo sapiens R-HSA-5617833	8/187	4.28%	1.3E-06	0.0004	YWHAE; CCT3; CCT2; DYNC1H1; PPP2R1A; TCP1; YWHAG; TUBA4A
2	Formation of tubulin folding intermediates by CCT/TriC Homo sapiens R-HSA-389960	4/25	16.00%	3.6E-06	0.0004	CCT3; CCT2; TCP1; TUBA4A
3	Prefoldin mediated transfer of substrate to CCT/TriC Homo sapiens R-HSA-389957	4/27	14.81%	4.9E-06	0.0004	CCT3; CCT2; TCP1; TUBA4A
4	Folding of actin by CCT/TriC Homo sapiens R-HSA-390450	3/10	30.00%	8.7E-06	0.0004	CCT3; CCT2; TCP1;
5	G2/M Transition Homo sapiens R-HSA-69275	7/173	4.05%	9.0E-06	0.0004	YWHAE; DYNC1H1; XPO1; RBBP4; PPP2R1A; YWHAG; TUBA4A
6	Mitotic G2-G2/M phases Homo sapiens R-HSA-453274	7/175	4.00%	9.7E-06	0.0004	YWHAE; DYNC1H1; XPO1; RBBP4; PPP2R1A; YWHAG; TUBA4A
7	Cooperation of Prefoldin and TriC/CCT in actin and tubulin folding Homo sapiens R-HSA-389958	4/32	12.50%	1.0E-05	0.0004	CCT3; CCT2; TCP1; TUBA4A;
8	Glycolysis Homo sapiens R-HSA-70171	4/32	12.50%	1.0E-05	0.0004	TPI1; PKM; PPP2R1A; PFKP
9	Organelle biogenesis and maintenance Homo sapiens R-HSA-1852241	9/326	2.76%	1.0E-05	0.0004	YWHAE; CCT3; CCT2; DYNC1H1; PPP2R1A; TCP1; MRPL16; YWHAG; TUBA4A
10	Loss of Nlp from mitotic centrosomes Homo sapiens R-HSA-380259	5/69	7.25%	1.1E-05	0.0004	YWHAE; DYNC1H1; PPP2R1A; YWHAG; TUBA4A
11	Loss of proteins required for interphase microtubule organization?from the centrosome Homo sapiens R-HSA-380284	5/69	7.25%	1.1E-05	0.0004	YWHAE; DYNC1H1; PPP2R1A; YWHAG; TUBA4A
12	AURKA Activation by TPX2 Homo sapiens R-HSA-8854518	5/72	6.94%	1.4E-05	0.0005	YWHAE; DYNC1H1; PPP2R1A; YWHAG; TUBA4A
13	Purine ribonucleoside monophosphate biosynthesis Homo sapiens R-HSA-73817	3/12	25.00%	1.6E-05	0.0005	ATIC; IMPDH2; PAICS
14	Glucose metabolism Homo sapiens R-HSA-70326	5/79	6.33%	2.2E-05	0.0006	TPI1; PKM; PPP2R1A; PGM1; PFKP;
15	Centrosome maturation Homo sapiens R-HSA-380287	5/79	6.33%	2.2E-05	0.0006	YWHAE; DYNC1H1; PPP2R1A; YWHAG; TUBA4A;
16	Recruitment of mitotic centrosome proteins and complexes Homo sapiens R-HSA-380270	5/79	6.33%	2.2E-05	0.0006	YWHAE; DYNC1H1; PPP2R1A; YWHAG; TUBA4A;
17	Regulation of PLK1 Activity at G2/M Transition Homo sapiens R-HSA-2565942	5/87	5.75%	3.5E-05	0.0009	YWHAE; DYNC1H1; PPP2R1A; YWHAG; TUBA4A;
18	Gene Expression Homo sapiens R-HSA-74160	19/1631	1.16%	4.3E-05	0.0010	YWHAE; DDX6; DARS; NSUN2; DDX1; RTCB; CSTF2; RPSA; EPRS; ELAVL1; XPO1; DDX39A; HNRNPK TRIM28; RBBP4; PPP2R1A; GARS; IGF2BP3; YWHAG
19	Anchoring of the basal body to the plasma membrane Homo sapiens R-HSA-5620912	5/97	5.15%	5.8E-05	0.0013	YWHAE; DYNC1H1; PPP2R1A; YWHAG; TUBA4A;
20	tRNA processing Homo sapiens R-HSA-72306	5/103	4.85%	7.8E-05	0.0016	NSUN2; DDX1; RTCB; CSTF2; EPRS;
21	BBSome-mediated cargo-targeting to cilium Homo sapiens R-HSA-5620922	3/23	13.04%	1.2E-04	0.0025	CCT3; CCT2; TCP1
22	Cytosolic tRNA aminoacylation Homo sapiens R-HSA-379716	3/24	12.50%	1.4E-04	0.0027	DARS; GARS; EPRS
23	Antigen Presentation: Folding, assembly and peptide loading of class I MHC Homo sapiens R-HSA-983170	3/25	12.00%	1.6E-04	0.0029	PDIA3; CANX; HLA-B
24	Metabolism Homo sapiens R-HSA-1430728	19/1908	1.00%	3.4E-04	0.0059	FDPS; DARS; TPI1; RPSA; EPRS; PGD; PAICS; ATP5J2; SRM; ALDH3A2; ATIC; PKM; MTHFD1; PPP2R1A; IMPDH2; UQCRC1; CKB; PGM1; PFKP
25	Purine metabolism Homo sapiens R-HSA-73847	3/34	8.82%	4.0E-04	0.0067	ATIC; IMPDH2; PAICS
26	HuR (ELAVL1) binds and stabilizes mRNA Homo sapiens R-HSA-450520	2/8	25.00%	4.9E-04	0.0079	XPO1; ELAVL1
27	Association of TriC/CCT with target proteins during biosynthesis Homo sapiens R-HSA-390471	3/39	7.69%	6.1E-04	0.0094	CCT3; CCT2; TCP1
28	Chaperonin-mediated protein folding Homo sapiens R-HSA-390466	4/95	4.21%	7.2E-04	0.0103	CCT3; CCT2; TCP1; TUBA4A;
29	Cooperation of PDCL (PhLP1) and TRiC/CCT in G-protein beta folding Homo sapiens R-HSA-6814122	3/42	7.14%	7.5E-04	0.0103	CCT3; CCT2; TCP1
30	tRNA Aminoacylation Homo sapiens R-HSA-379724	3/42	7.14%	7.5E-04	0.0103	DARS; GARS; EPRS
31	RHO GTPase Effectors Homo sapiens R-HSA-195258	6/255	2.35%	7.6E-04	0.0103	YWHAE; XPO1; PPP2R1A; PRC1; CTNNA1; YWHAG
32	Protein folding Homo sapiens R-HSA-391251	4/101	3.96%	9.1E-04	0.0118	CCT3; CCT2; TCP1; TUBA4A;
33	SeMet incorporation into proteins Homo sapiens R-HSA-2408517	2/11	18.18%	9.6E-04	0.0121	DARS; EPRS
34	Chk1/Chk2(Cds1) mediated inactivation of Cyclin B:Cdk1 complex Homo sapiens R-HSA-75035	2/12	16.67%	1.1E-03	0.0141	YWHAE; YWHAG
35	Metabolism of carbohydrates Homo sapiens R-HSA-71387	6/282	2.13%	1.3E-03	0.0153	TPI1; PKM; PPP2R1A; PGD; PGM1; PFKP
36	Cargo trafficking to the periciliary membrane Homo sapiens R-HSA-5620920	3/51	5.88%	1.3E-03	0.0155	CCT3; CCT2; TCP1
37	Calnexin/calreticulin cycle Homo sapiens R-HSA-901042	2/15	13.33%	1.8E-03	0.0197	PDIA3; CANX
38	Activation of BAD and translocation to mitochondria Homo sapiens R-HSA-111447	2/15	13.33%	1.8E-03	0.0197	YWHAE; YWHAG
39	tRNA processing in the nucleus Homo sapiens R-HSA-6784531	3/57	5.26%	1.8E-03	0.0197	DDX1; RTCB; CSTF2
40	Metabolism of proteins Homo sapiens R-HSA-392499	12/1074	1.12%	1.9E-03	0.0198	PDIA3; CCT3; CCT2; DYNC1H1; HNRNPK; TCP1; CANX; RPSA; SPTAN1; CKAP4; TUBA4A; SPTBN1;
41	N-glycan trimming in the ER and Calnexin/Calreticulin cycle Homo sapiens R-HSA-532668	2/17	11.76%	2.3E-03	0.0237	PDIA3; CANX
42	Influenza Life Cycle Homo sapiens R-HSA-168255	4/136	2.94%	2.7E-03	0.0269	XPO1; CANX; RPSA; IPO5;
43	Cell Cycle, Mitotic Homo sapiens R-HSA-69278	7/462	1.52%	3.5E-03	0.0340	YWHAE; DYNC1H1; XPO1; RBBP4; PPP2R1A; YWHAG; TUBA4A
44	Influenza Infection Homo sapiens R-HSA-168254	4/147	2.72%	3.6E-03	0.0340	XPO1; CANX; RPSA; IPO5;
45	Nephrin interactions Homo sapiens R-HSA-373753	2/22	9.09%	3.9E-03	0.0355	SPTAN1; SPTBN1
46	Cyclin A/B1 associated events during G2/M transition Homo sapiens R-HSA-69273	2/22	9.09%	3.9E-03	0.0355	XPO1; PPP2R1A
47	COPI-mediated anterograde transport Homo sapiens R-HSA-6807878	3/78	3.85%	4.5E-03	0.0398	DYNC1H1; SPTAN1; SPTBN1
48	Signaling by Rho GTPases Homo sapiens R-HSA-194315	6/367	1.63%	4.7E-03	0.0412	YWHAE; XPO1; PPP2R1A; PRC1; CTNNA1; YWHAG
49	Cytokine Signaling in Immune system Homo sapiens R-HSA-1280215	8/620	1.29%	4.8E-03	0.0412	PPP2R1A; HLA-B; PHB; IFIT1; SPTAN1; TRIM21; SQSTM1; SPTBN1

The analysis was performed using the EnrichR app and the Reactome database. Each hit is additionally supplemented with proteins enriching the given ontological term.

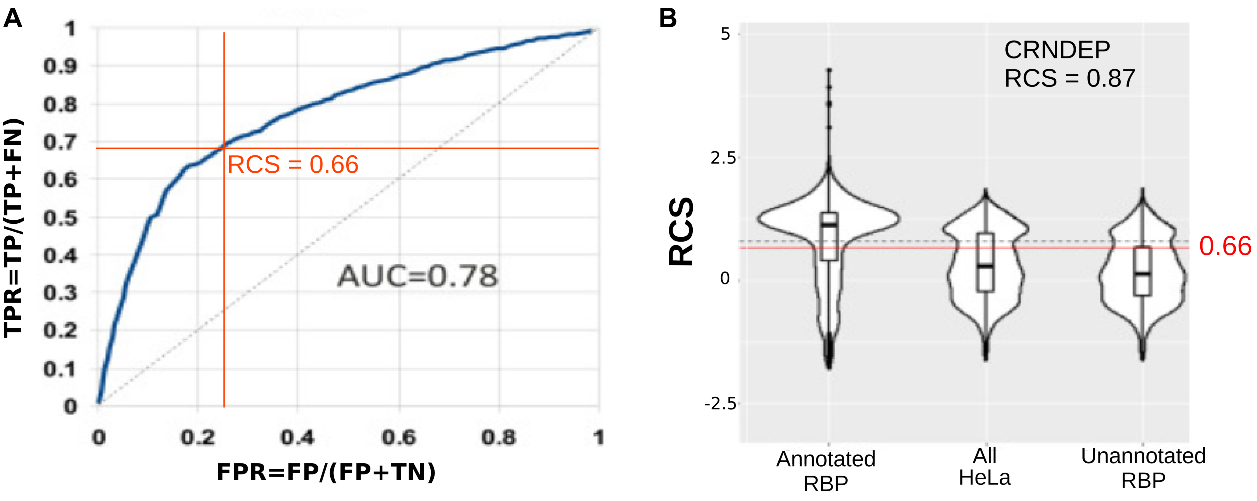

Fig. S18. Assessment of RNA binding capabilities of CRNDEP. The ability of CRNDEP to bind RNA molecules was evaluated with the SONAR application. The ROC curve plotted for the generated statistical model (A) revealed its good discriminating capabilities. The model was able to distinguish RNA-binding from non-biding proteins with the sensitivity (TPR) and specificity (1-FPR) of 0.69 and 0.74, respectively (RCS cut-off point = 0.66, AUC = 0.78). The RCS value estimated with the same model for CRNDEP was 0.87 (denoted with a gray dashed line in B) which implies that CRNDEP can probably bind RNA molecules. RBP – RNA-binding protein, RCS – RBP classification score, Annotated RBP – all identified RBPs in the HeLa cell line, All HeLa – a whole protein set from the HeLa cell line, Unannotated RBP – proteins from the HeLa cell line not being RBPs, TPR – true positive rate, FPR – false positive rate, FP – false positive, TN – true negative, TP – true positive, FN – false negative, AUC – area under ROC curve.

Analysis of relative *CRNDE* transcripts expression level in different ovarian cancer cell lines.

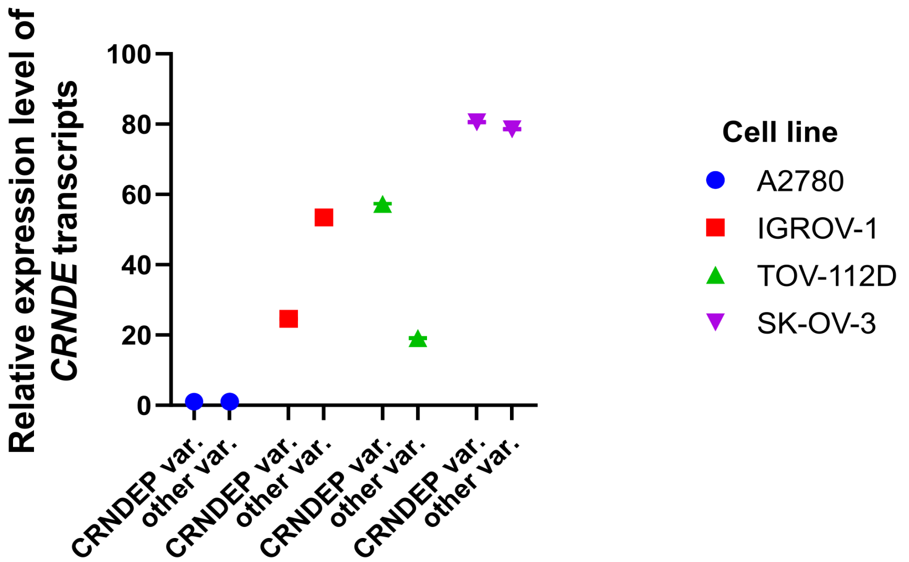

Fig. S19. Relative *CRNDE* expression in different ovarian cancer cell lines. Comparative expression analysis was performed for different *CRNDE* splice variants (transcripts): CRNDEP var. – transcripts coding for CRNDEP micropeptide (FJ466686.1 and NR_170995.1), other var. – other CRNDEP-non-coding transcripts (FJ466685.1, NR_034105.4 and NR_034106.3). *CRNDE* expression was normalized against the expression of *the HGPRT* reference gene. The A2780 cell line was used as a calibrator.

Table S10. A list of synthetic DNA molecules utilized in the present study.

Molecule name	Sequence (5' → 3')	Description
SH1_CRNDE_Top	GATCGGGAGCTACTGTACTGGCTATTGGAAGGAGTCAAGAGCTCCTTCCAATAGCCAGTACAGTAGCTCCTTTTTTGA	Pair of mutually complementary oligonucleotides forming hairpins after their denaturation and subsequent renaturation. Sticky ends specific to HindIII and BamHI restriction enzymes are underlined. This hairpin is capable of silencing the expression of all reference *CRNDE* transcripts, i.e., NR_034105.4, NR_034106.3, NR_110453.2, NR_110454.2, NR_170995.1.
SH1_CRNDE_Bot	AGCTTCAAAAAAGGAGCTACTGTACTGGCTATTGGAAGGAGCTCTTGACTCCTTCCAATAGCCAGTACAGTAGCTCCC	
SH2_CRNDE_Top	GATCGAGAAGAAGGTTAAGCTGTATTTGATTGCCTCAAGAGGGCAATCAAATACAGCTTAACCTTCTTCTTTTTTTGA	Pair of mutually complementary oligonucleotides forming hairpins after their denaturation and subsequent renaturation. Sticky ends specific to HindIII and BamHI restriction enzymes are underlined. This hairpin is capable of silencing the expression of all reference *CRNDE* transcripts, i.e., NR_034105.4, NR_034106.3, NR_110453.2, NR_110454.2, NR_170995.1.
SH2_CRNDE_Bot	AGCTTCAAAAAAAGAAGAAGGTTAAGCTGTATTTGATTGCCCTCTTGAGGCAATCAAATACAGCTTAACCTTCTTCTC	
SH3_CRNDE_Top	GATCGGAAGATAAGGAGGATGCCACTGGAAATGTTCAAGAGACATTTCCAGTGGCATCCTCCTTATCTTCTTTTTTGA	Pair of mutually complementary oligonucleotides forming hairpins after their denaturation and subsequent renaturation. Sticky ends specific to HindIII and BamHI restriction enzymes are underlined. This hairpin is capable of silencing the expression of the CRNDEP-coding reference transcript only, i.e. NR_170995.1.
SH3_CRNDE_Bot	AGCTTCAAAAAAGAAGATAAGGAGGATGCCACTGGAAATGTCTCTTGAACATTTCCAGTGGCATCCTCCTTATCTTCC	
SCR_Top	GATCGGGAGCAATATCGTGGATGAAACGGTGAAATCAAGAGTTTCACCGTTTCATCCACGATATTGCTCCTTTTTTGA	Pair of mutually complementary oligonucleotides forming hairpins after their denaturation and subsequent renaturation. Sticky ends specific to HindIII and BamHI restriction enzymes are underlined. This scrambled (control) hairpin is incapable of silencing any known human transcripts.
SCR_Bot	AGCTTCAAAAAAGGAGCAATATCGTGGATGAAACGGTGAAACTCTTGATTTCACCGTTTCATCCACGATATTGCTCCC	
Bglob-pA-R	CCCATATGTCCTTCCGAGTG	Sequencing primer
CAG_seqF	GCCTCTGCTAACCATGTTC	PCR primer used for amplification of the Flagx2 insert.
FL243mR	CCGAATTCaCTTGTCATCG	Primer used in a combination with the CAG_seqF primer for amplification of the Flagx2 insert. The lowercase adenine was introduced to the original Flagx2-coding sequence to generate a stop codon after the Flag-tags.
sgRNA_AAVS1_TOP	ACCGGGGCCACTAGGGACAGGAT	Pair of oligonucleotides which after denaturation and renaturation form the DNA fragment encoding the sgRNA T2, that targets the Cas9 endonuclease to the AAVS1 safe harbor. BbsI-specific sticky ends are
sgRNA_AAVS1_BOT	AAACATCCTGTCCCTAGTGGCCC	

Molecule name	Sequence (5’ → 3’)	Description
		underlined.
primer_B	GGGGACAAGTTTGTACAAAAAAGCAGGCTTCACCATGTTGGCTGAAATTCATCCCAAGG	Primers B and L were used to amplify the CRNDEP ORF sequence encompassed by the attB sites, allowing for the BP clonase-catalyzed recombination of the obtained PCR product into the pDONR vector used in the Gateway cloning system (Thermo Fisher Scientific). The Kozak sequence is underlined.
primer_L	GGGGACCACTTTGTACAAGAAAGCTGGGTGTAGTCTATAAACAGGAATACCC	
promoU6_F	GGACTATCATATGCTTACCGT	Sequencing primer complementary to the U6 promoter
SV40	GTGGTTTGTCCAAACTCATC	Sequencing primer complementary to the replication origin of the SV40 virus.

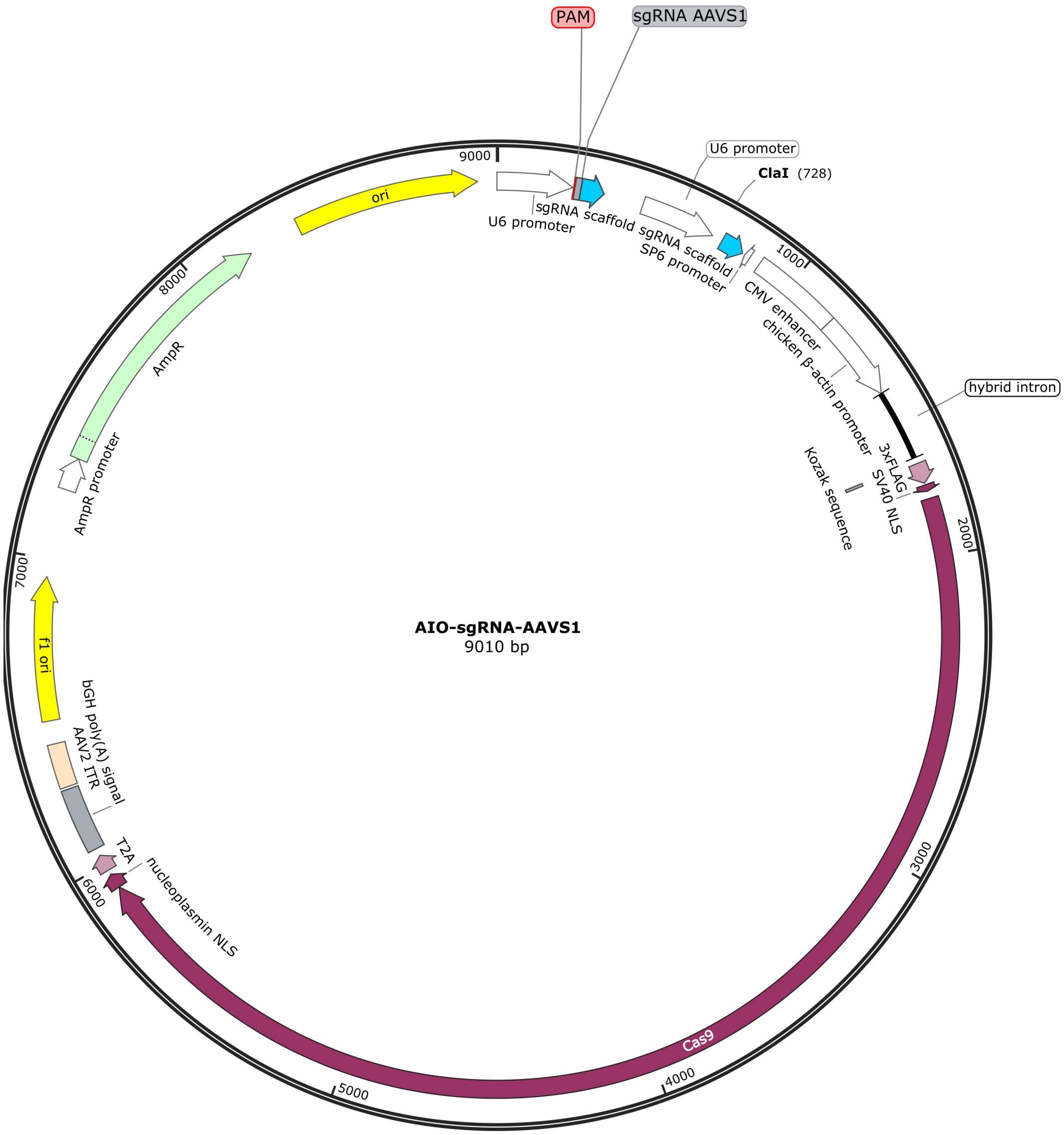

Fig. S20. AIO-sgRNA-AAVS1 plasmid scheme. This construct was used for CRISPR/Cas9-dependent editing of the SK-OV-3 and A2780 ovarian cancer cell lines' genomes. It encodes a mutated Cas9 variant (D10A) which cuts only one strand of genomic DNA. The plasmid additionally codes for the AAVS1 locus-targeting sgRNA expressed from the U6 promoter. sgRNA – single-guide RNA. PAM – protospacer adjacent motif.

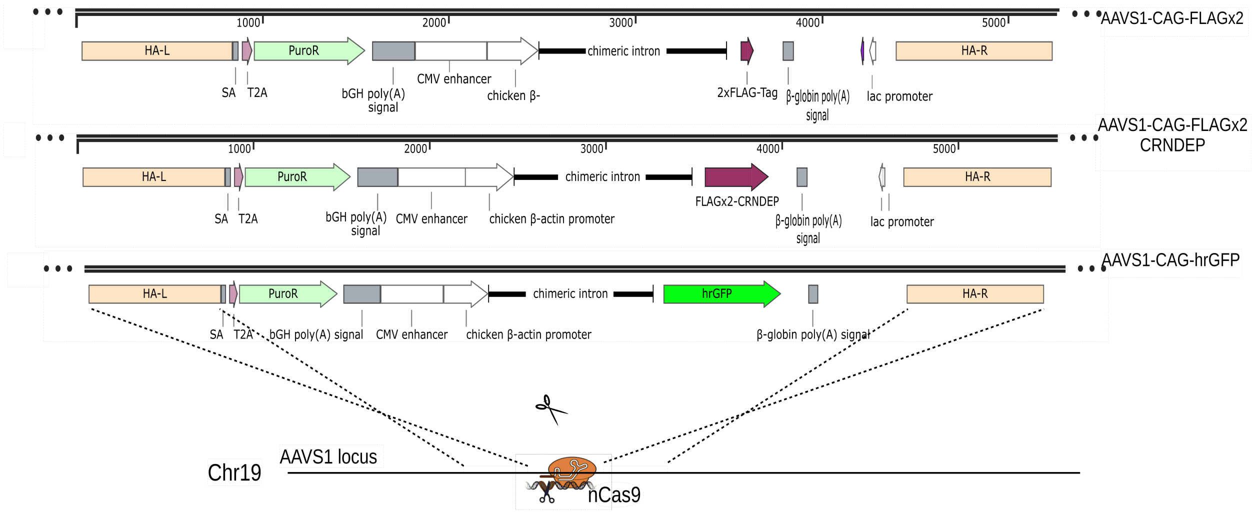

Fig. S21. A scheme of expression cassettes inserted into AAVS1 safe harbor locus in the SK-OV-3 and A2780 cell lines. Names of the plasmids harboring each cassette are provided on the right. All inserts contained puromycin resistance gene (PuroR), expression of which enabled the selection of cells with the cassettes integrated into their genomes.

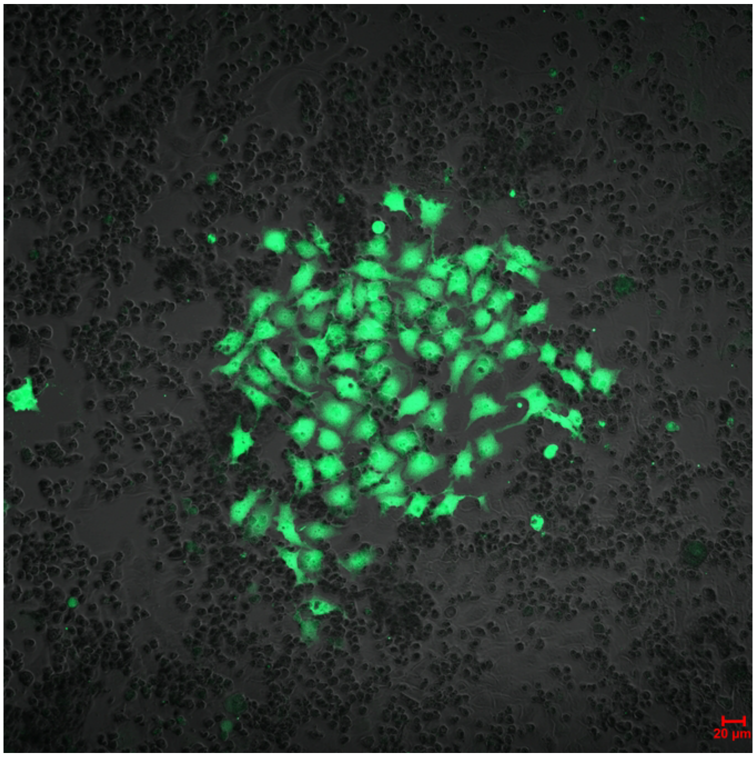

Fig. S22. A positive result of the *hrGFP* gene recombination into the HeLa genome using the CRISPR/Cas9 technology.

Table S11. A list of antibodies used in the present study.

#	Detected protein	Immunized species	Manufacturer	Catalog no.	Concentration /amount used (method)	Antibody type/ conjugate
1	CRNDEP	Rabbit	Abgent Inc.	custom-made	1:500 (IF, WB);<br>1:100 (DB);<br>10 µg (IP)	I/-
2	None (control IgG)	Rabbit	Sigma-Aldrich	I5006	10 µg (IP)	I/-
3	Paxillin	Rabbit	Abcam	abY113	1:250 (IF)	I/-
4	Phospho-Histone H3 (Ser10)*, pHH3	Mouse	Thermo	MA515220	1:1,000 (IF)	I/-
5	Flag-tag (clone M2)	Mouse	Sigma-Aldrich	A8592	1:1,500 (WB)	I/HRP
6	Flag-tag (clone M2)	Mouse	Sigma-Aldrich	F1804	1:1,000 (IF)	I/-
7	GFP	Mouse	Thermo	MA5-15256	1:500 (WB)	I/-
8	Centrin (clone 20H5)	Mouse	Merck Millipore	04-1624	1:500 (WB)	I/-
9	α-tubulin (clone DM1A)	Mouse	Thermo	62204	1:500 (WB)	I/-
10	GAPDH	Rabbit	CST	8884	1:500 (WB)	I/HRP
11	RFP	Rat	Proteintech	5f8	1:1,000 (WB)	I/-
12	Mouse IgG	Horse	Cell Signalling Technology	7076	1:10,000 (WB)	II/HRP
13	Mouse IgG	Goat	Thermo	A -11029	1:500 (IF)	II/Alexa Fluor 488
14	Mouse IgG	Goat	Thermo	A-21236	1:500 (IF)	II/Alexa Fluor 647
15	Rabbit IgG	Donkey	GE HealthCare	A934	1:10,000 (WB)	II/HRP
16	Rabbit IgG	Goat	Thermo	A-11034	1:500 (IF)	II/Alexa Fluor 488
17	Rabbit IgG	Goat	Thermo	A-21245	1:500 (IF)	II/Alexa Fluor 647
18	Rabbit IgG	Goat	Thermo	31462	1:10,000 (DB)	II/HRP
19	Rat IgG	Goat	Santa Cruz Biotechnology Inc.	sc-2006	1:10,000 (WB)	II/HRP

IF – immunofluorescence; WB – western blot; IP – immunoprecipitation; DB – dot blot; HRP – horseradish peroxidase; I – primary antibody; II – secondary antibody.

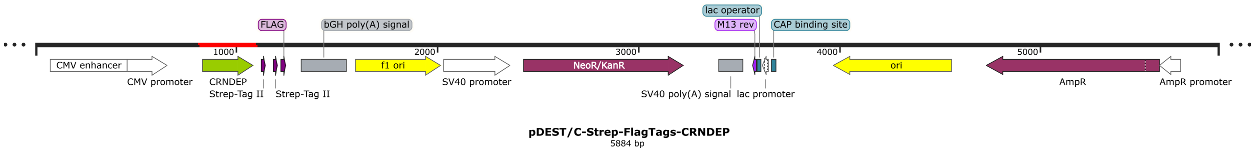

Fig. S23. A scheme of the pDEST/C-Strep-FlagTags-CRNDEP plasmid.

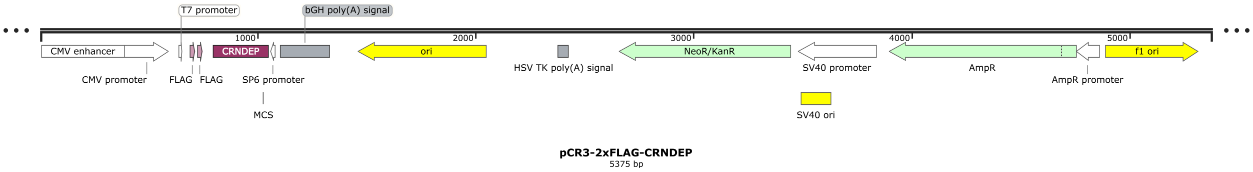

Fig. S24. A scheme of the pCR3-2xFLAG-CRNDEP plasmid. The FLAG2x-CRNDEP insert was subcloned from this plasmid to the AAVS1-CAG-hrGFP plasmid to get the AAVS1-CAG-FLAG2x-CRNDEP construct, harboring the AAVS1 locus-targeted expression cassette encoding the FLAG2x-CRNDEP fusion protein (see Fig. S21).

Table S12. DNA short tandem repeat (STR) profiling results.

Locus	Cell line				
	HeLa	SK-OV-3	A2780	IGROV-1	TOV-112D
Amelogenin	X	X	X	X	X
D3S1358	15, 18	14	14, 16, (17)	14, 15	15
D1S1656	12, 15	11, 17.3	12, 13	12, 13, (14), (15), (16)	18.3
D2S441	10, 11	10, 11.3	11, 12	11, 15	10, 14
D10S1248	13, 15	13, 16, (17)	13	12, 12	14
D13S317	(12), 13.3	8, 11	12, 13	8, 10	8
Penta E	7, 17	5, 13	10, 13	13, 17	11
D16S539	9, 10	12	11, 12, 13	11, 12	(9), (11), 12
D18S51	16	16, 17, (18)	(14), 16, 18, 19	15, 16	17
D2S1338	17	18, 23	21, 22	17, 25	19, 24
CSF1PO	9, 10	11	10, 11	11, (13), (14), (15)	12
Penta D	8, 15	12, 13	8, 9	8, 10	9
TH01	7	9, 9.3	6	7, 9.3	6
vWA	16, 18	17, 18	15, 16	16, (17), (20), (21), (22)	18
D21S11	27, 28	30, 31.2	28	26, (27), 30.2	31
D7S820	8, 12	13, 14	10	10.1, 10.3, (11.1)	9, 10
D5S818	11, 12	11	11, 12	(11), 12	10
TPOX	8, 12	8, 11	8, 10	8, 11	8, 11
D8S1179	12, 13	14, 15	15, 17, (18)	14, (15), 16	9, 13
D12S391	20, 25	22	19, 20	20, (25), (26), (27)	18, 18.3
D19S433	13, 14	14, 14.2	12	13, 14	14
SE33	20	16, 19	(18), 19, 25.2	(22), 23, (23.2), 24	19
D22S1045	16, 17	11, 16	15, 16	15, 16, 17, 18	11
FGA	18, 21	24, 25	19, 24	(20), 21, 25, 26	20

Alleles, the presence of which is uncertain due to weak fluorescence signals, are shown in brackets. Detailed results of the DNA STR profiling can be found in the supplementary file: Cell_lines_DNA_STR_profiling_results.pdf. The Y chromosome-specific loci (DYS391, DYS576 and DYS570) have been excluded from the table as no alleles in these loci were identified in any of the analyzed cell lines.
