# Supplementary figures and images for: A Multi-Faceted Analysis Showing CRNDE Transcripts and a Recently Confirmed Micropeptide as Important Players in Ovarian Carcinogenesis
Source: Int J Mol Sci. 2024 Apr 16;25(8):4381. doi: 10.3390/ijms25084381 (PMC11050281; doi:10.3390/ijms25084381)

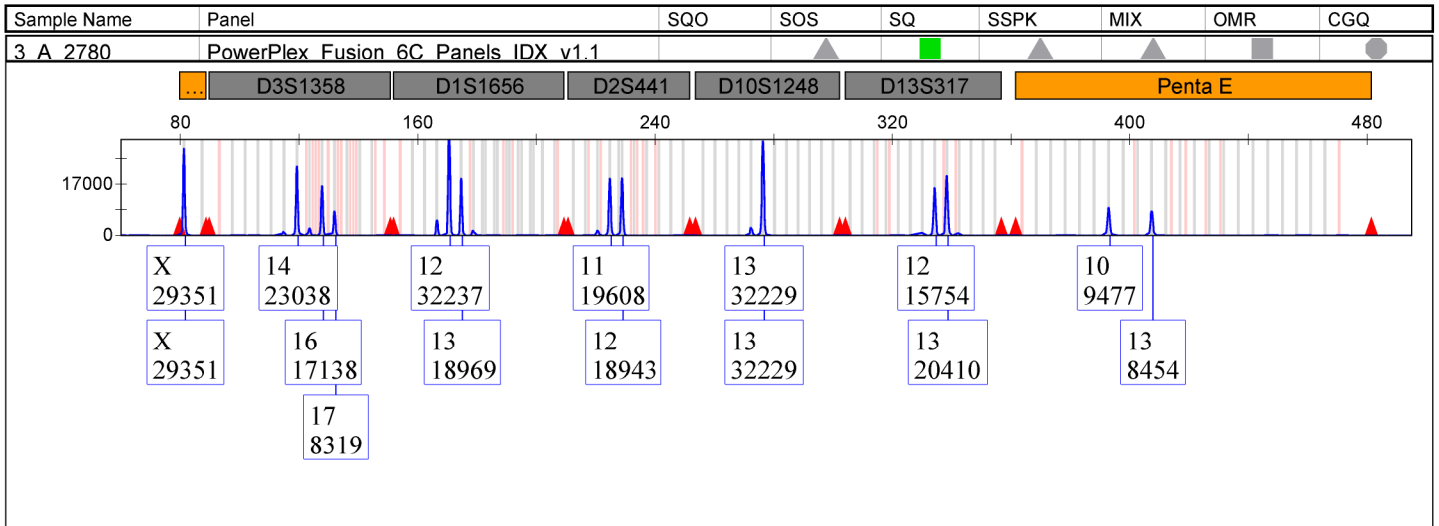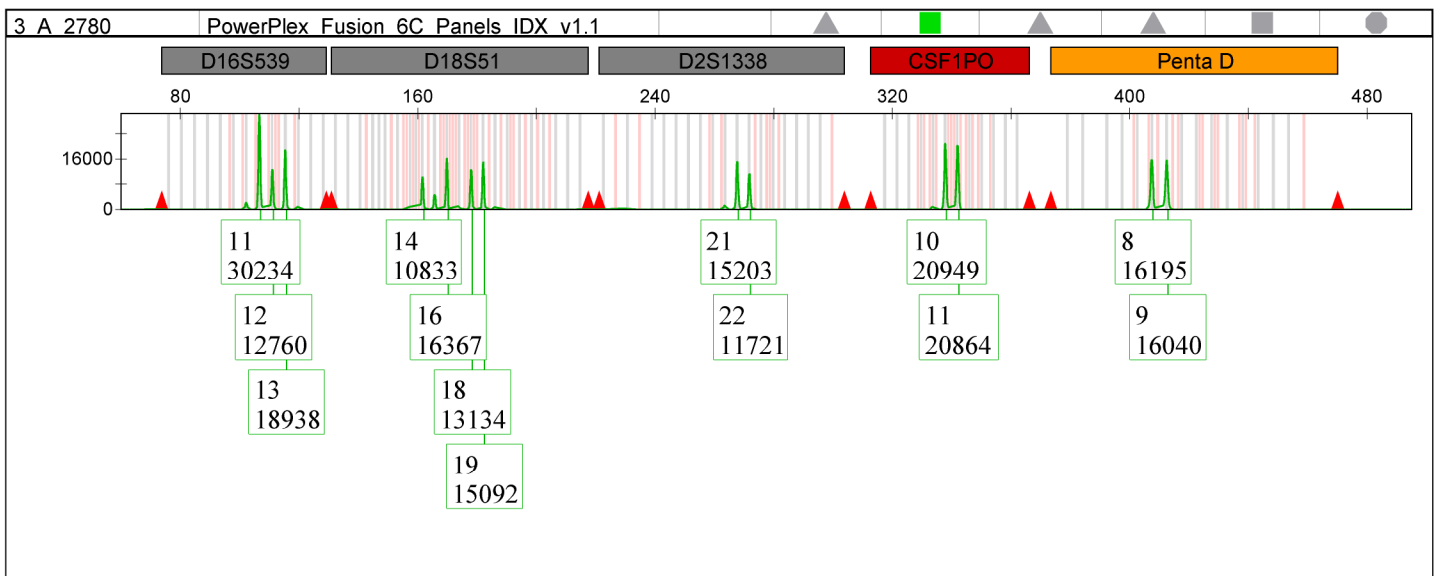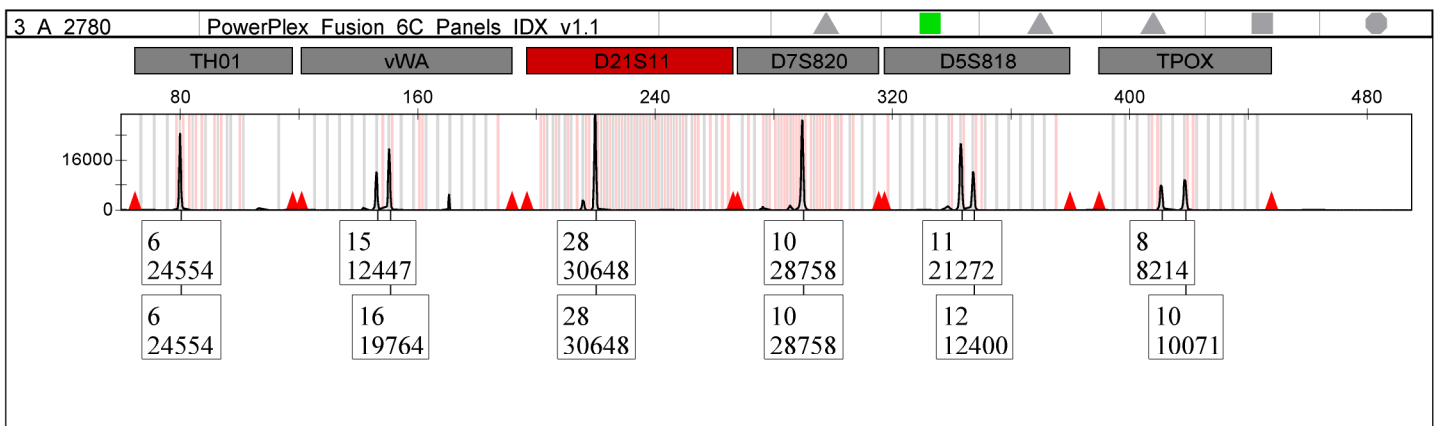

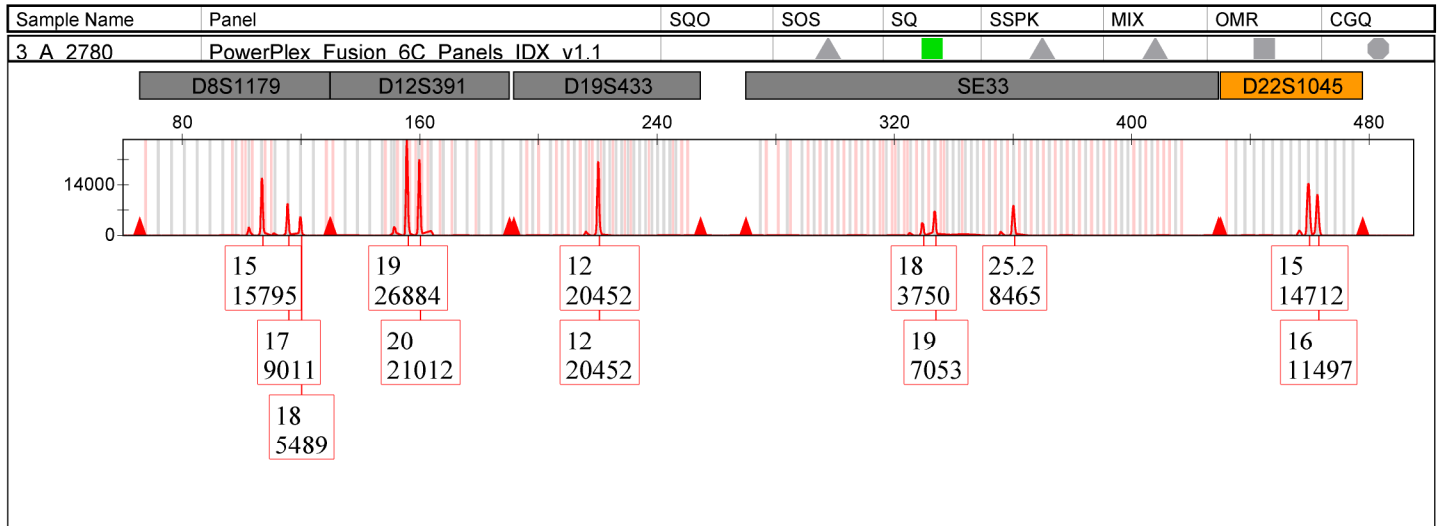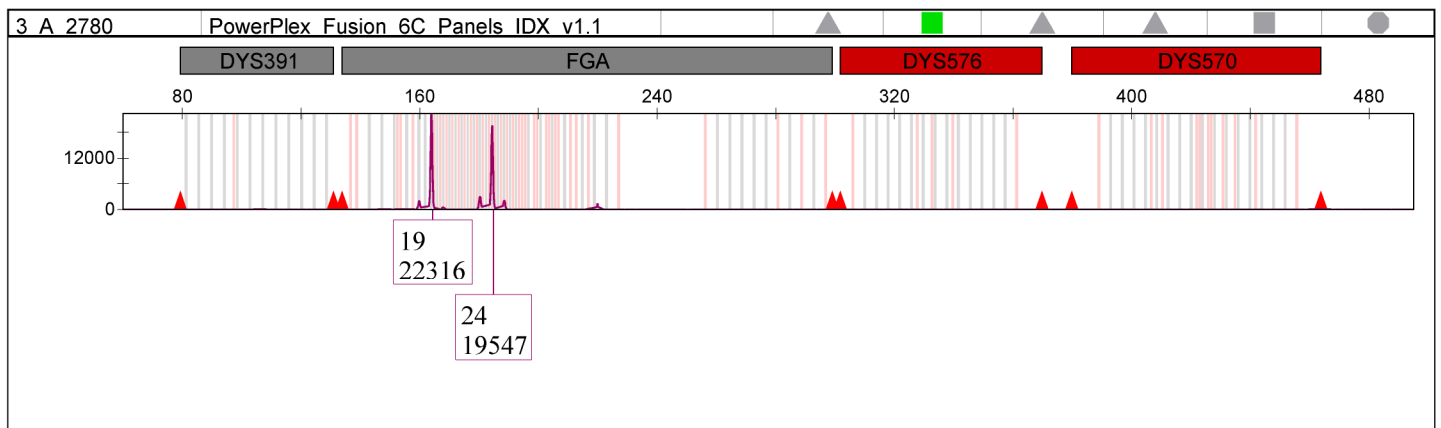

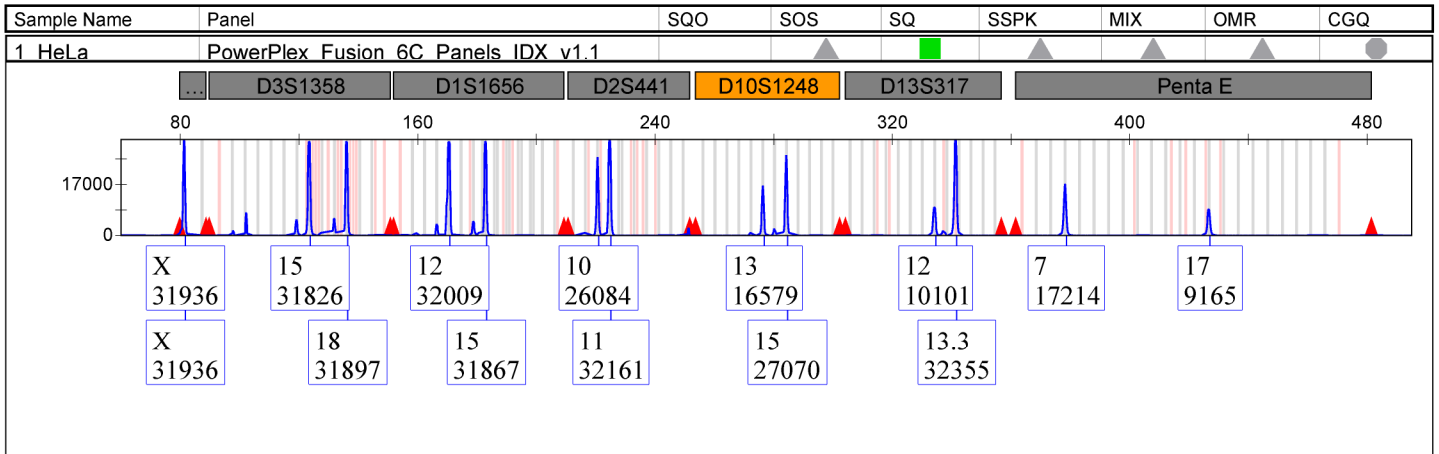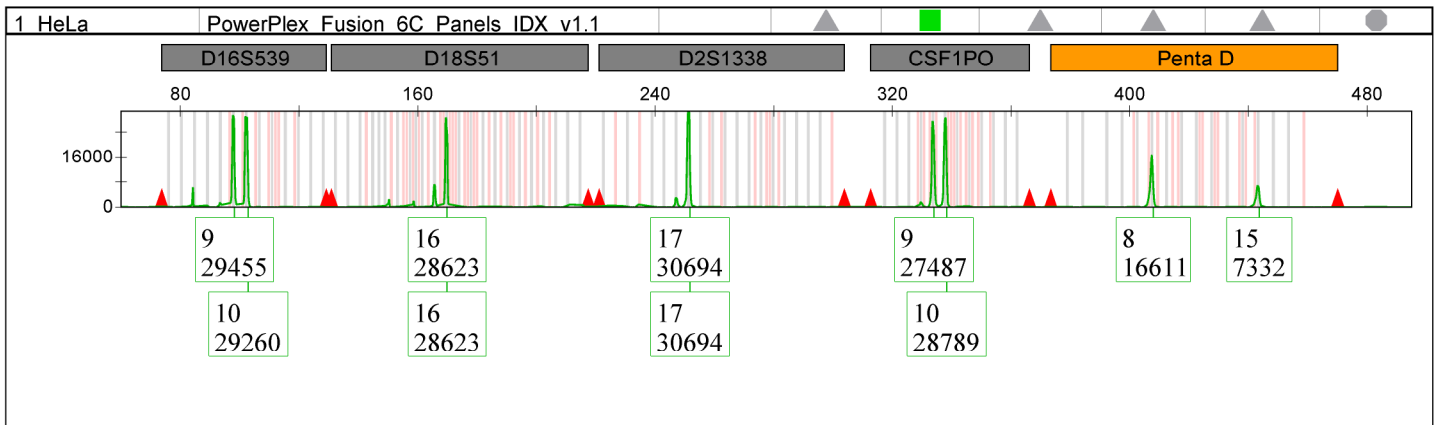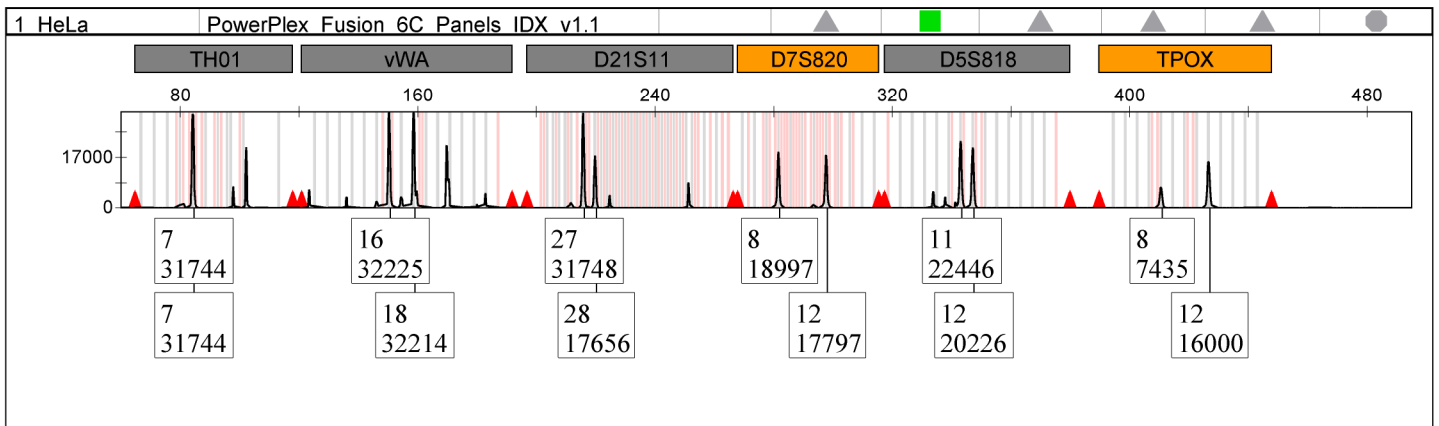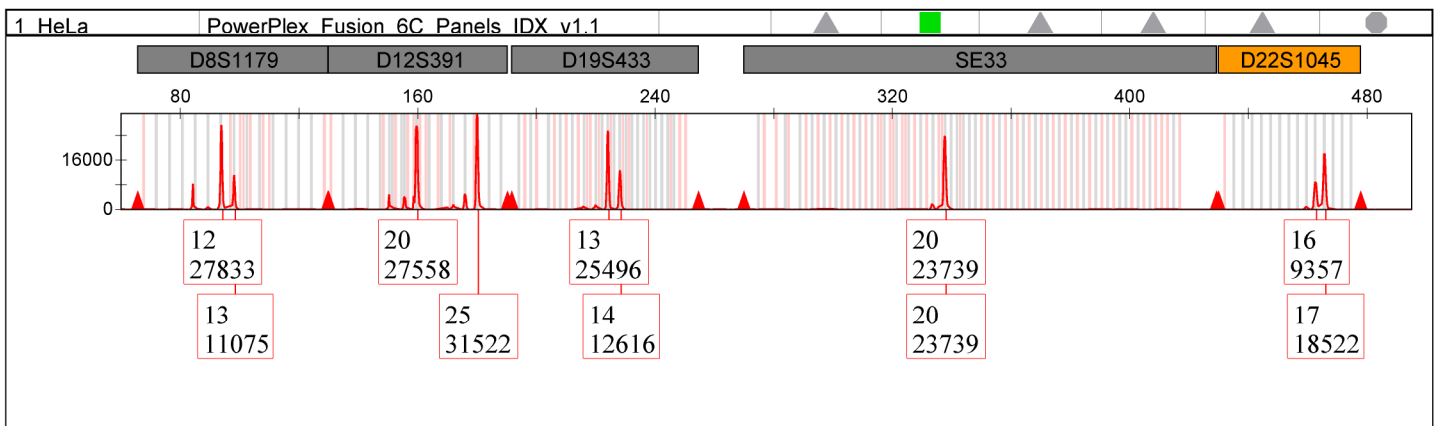

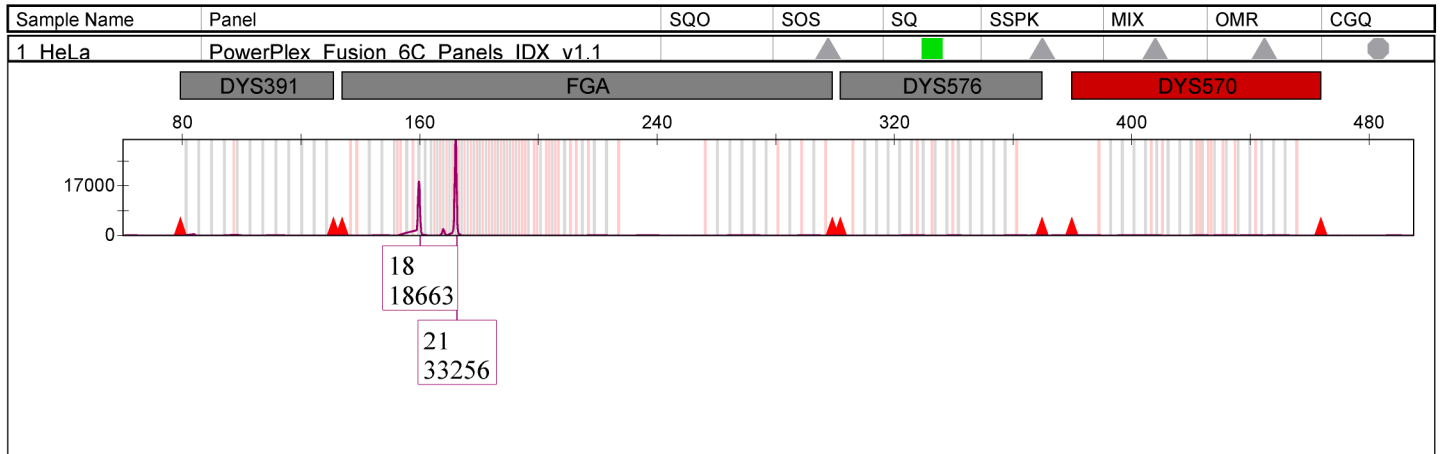

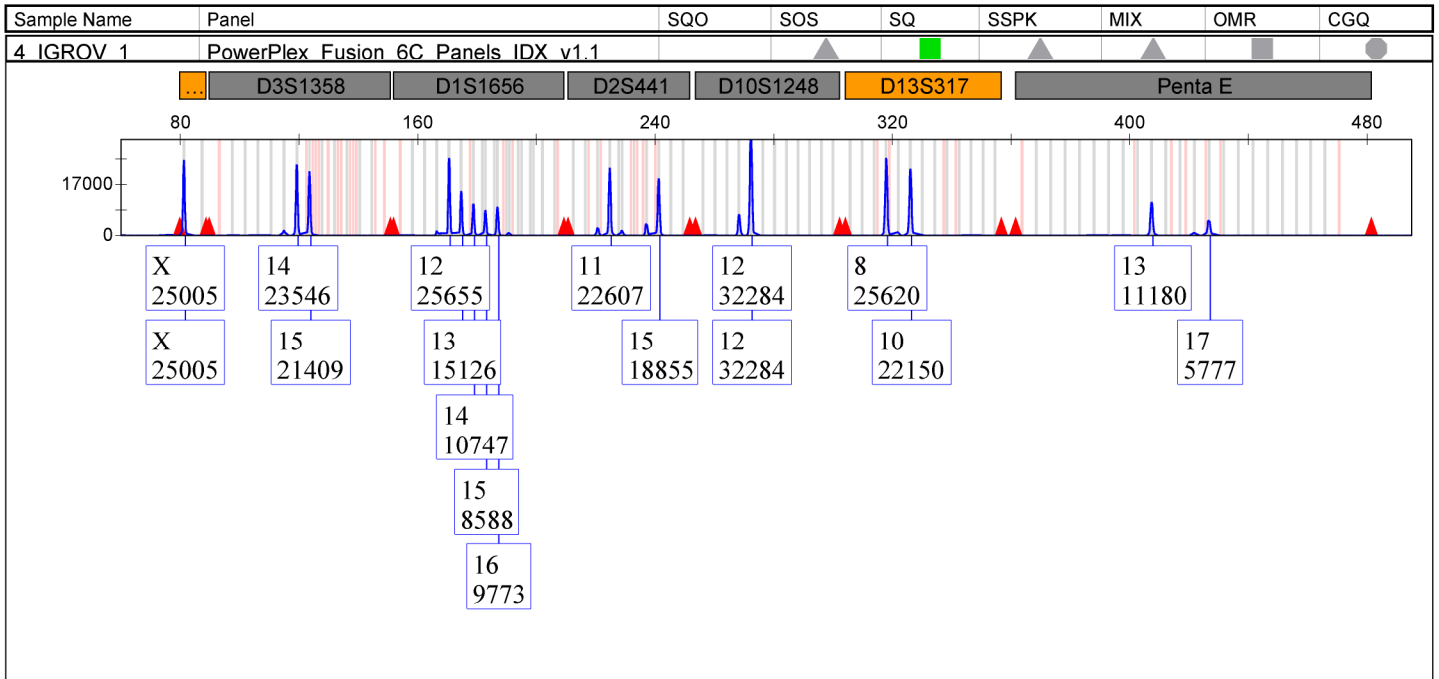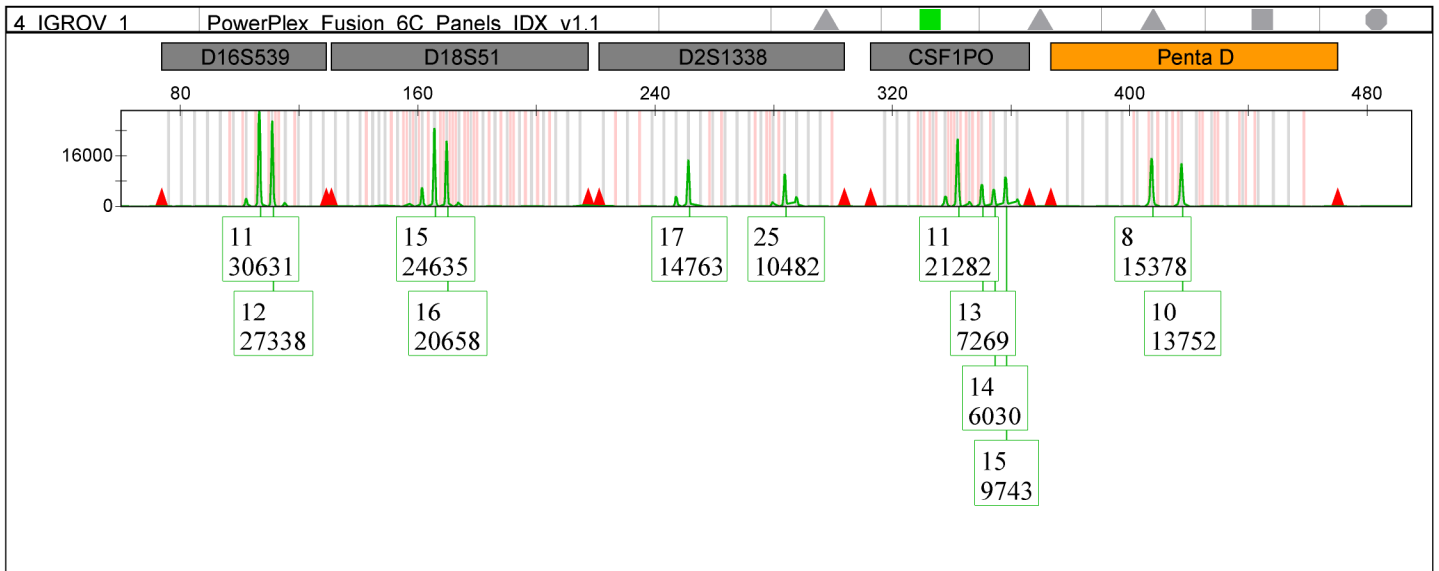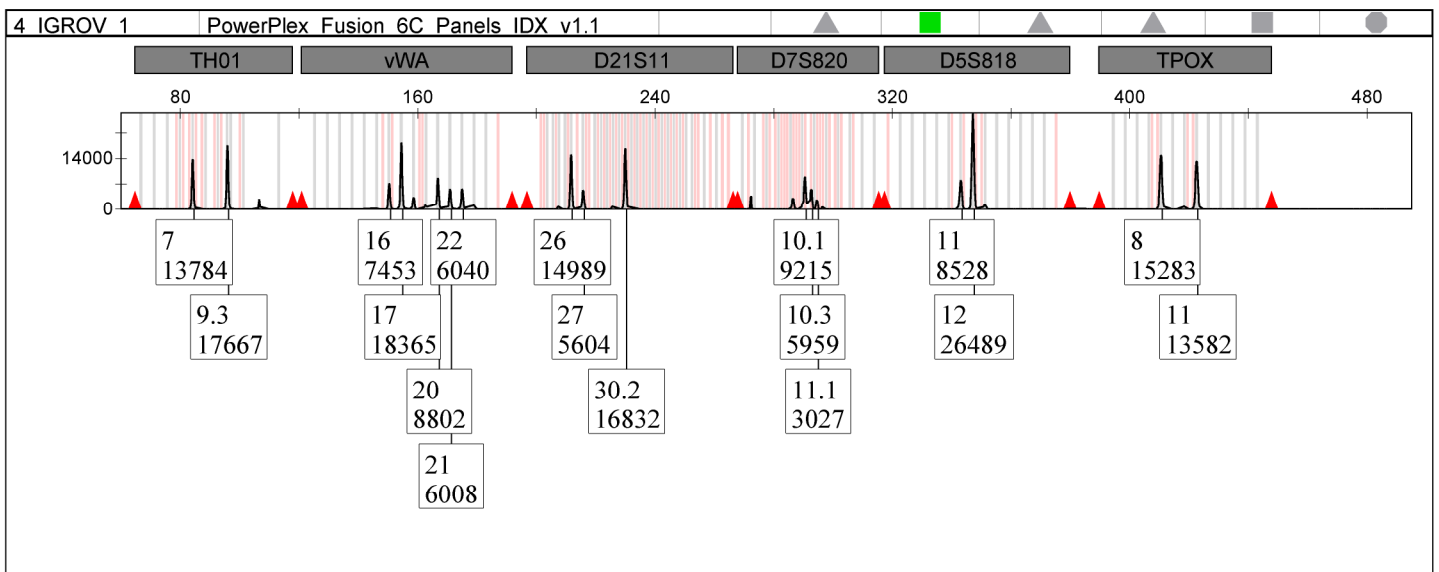

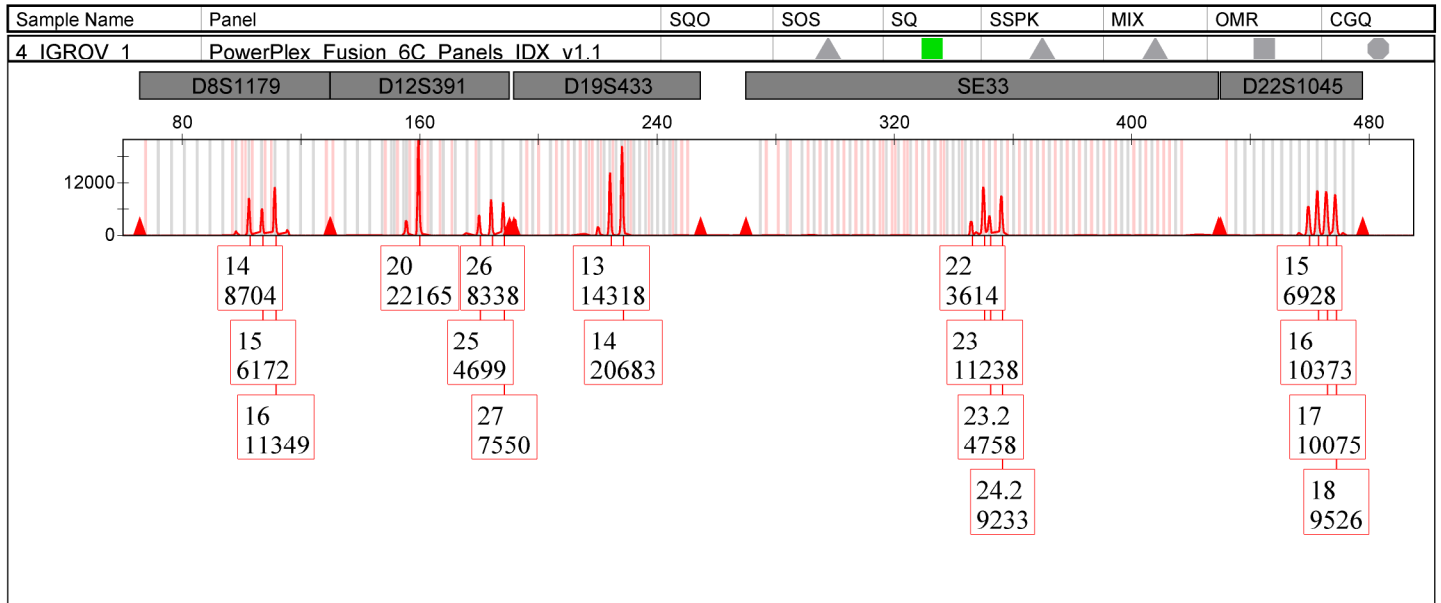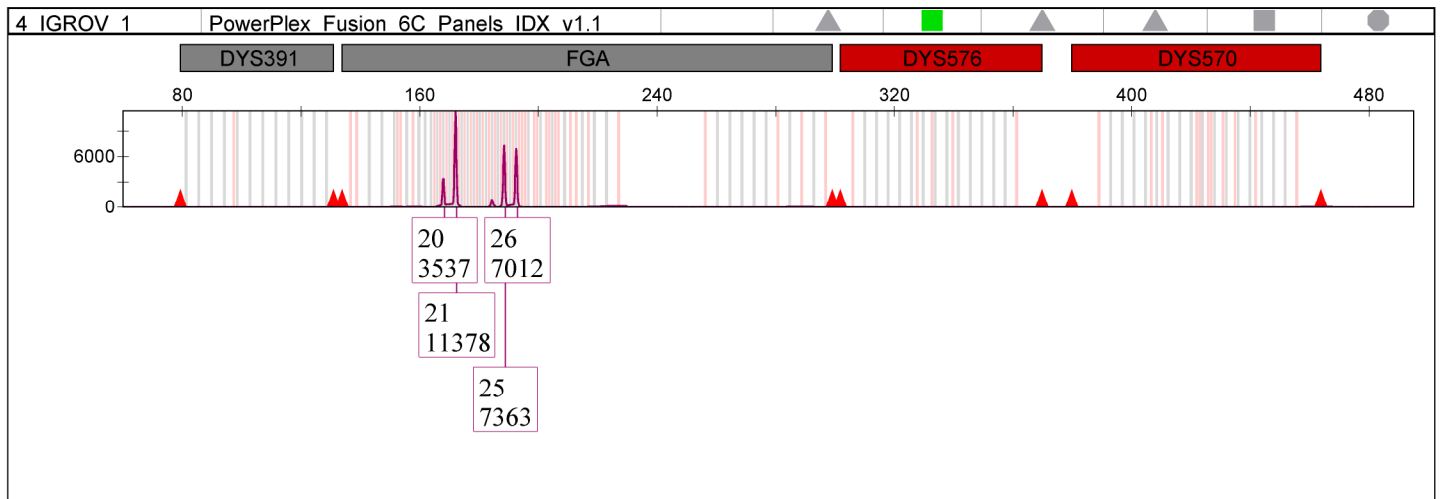

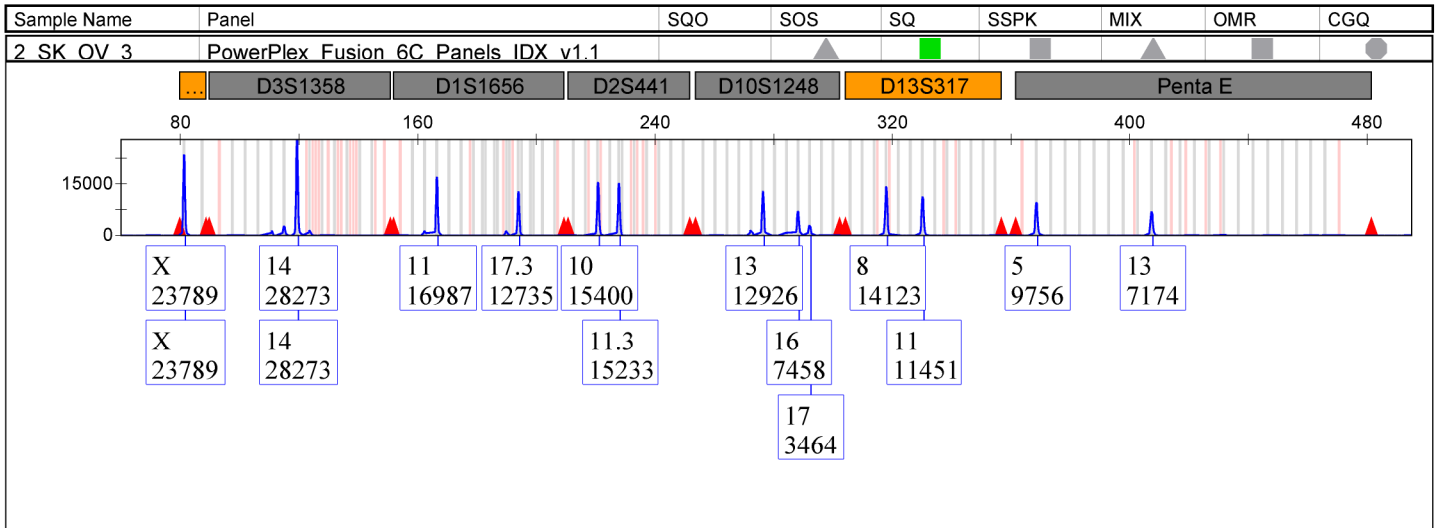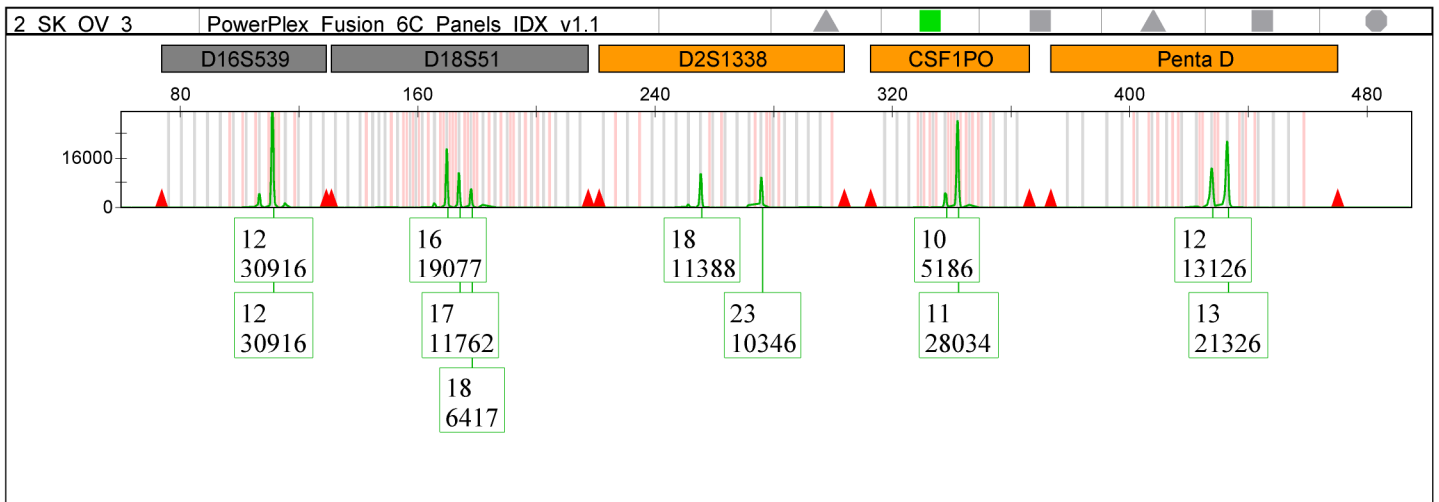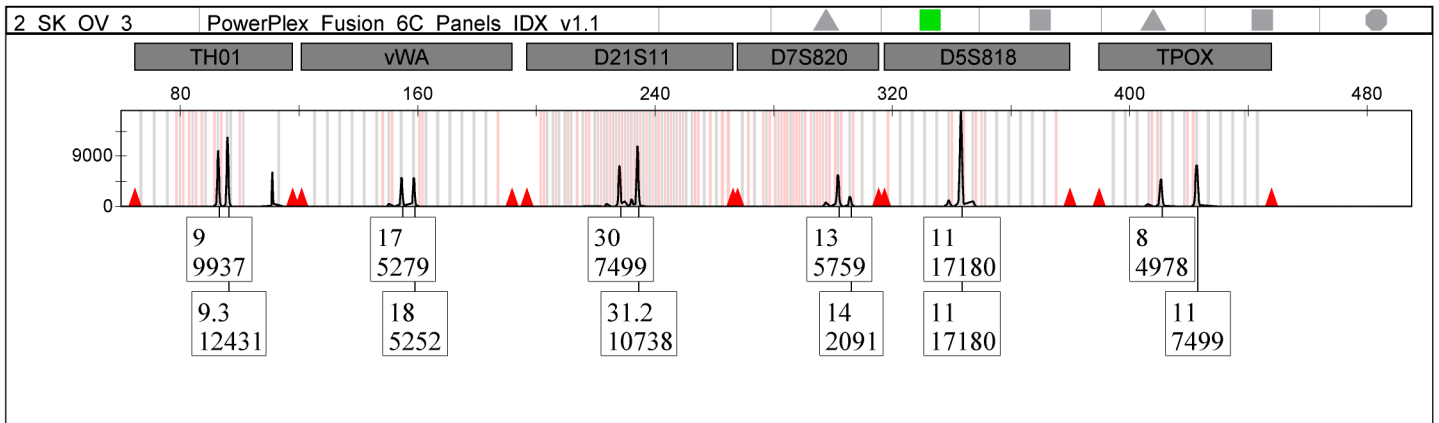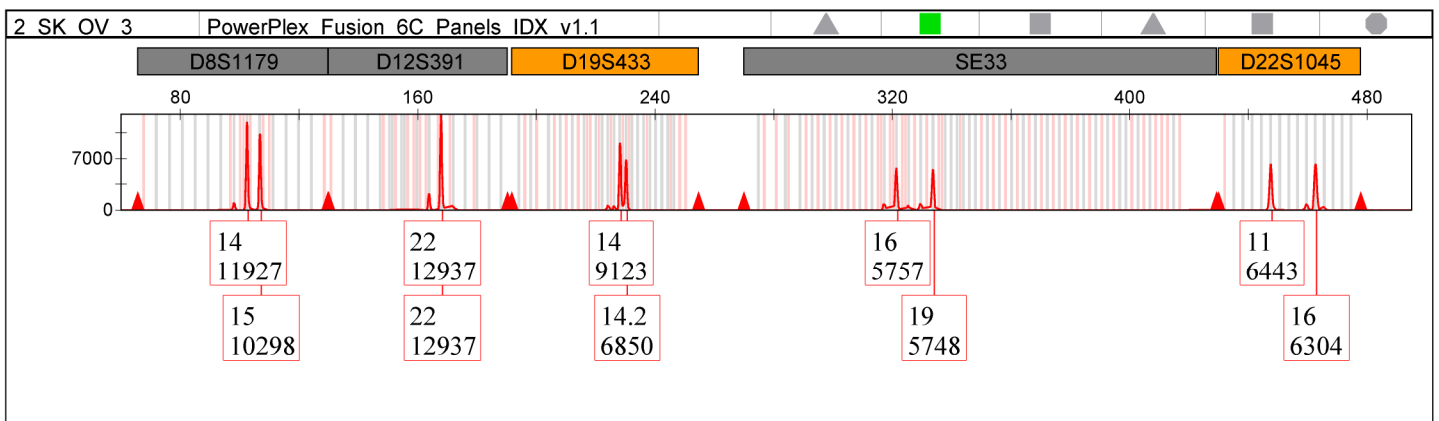

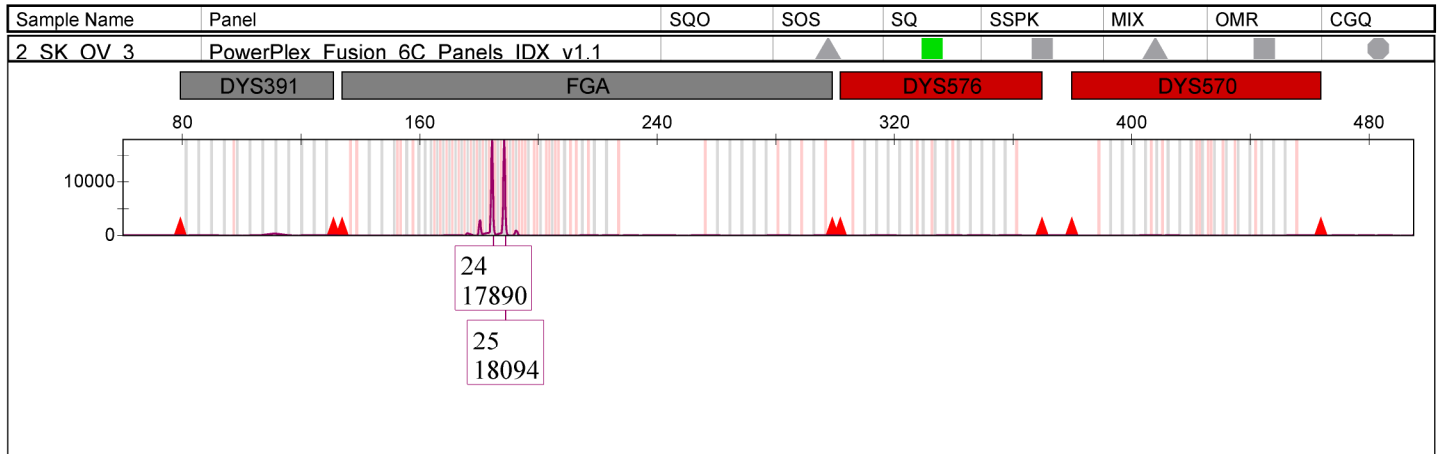

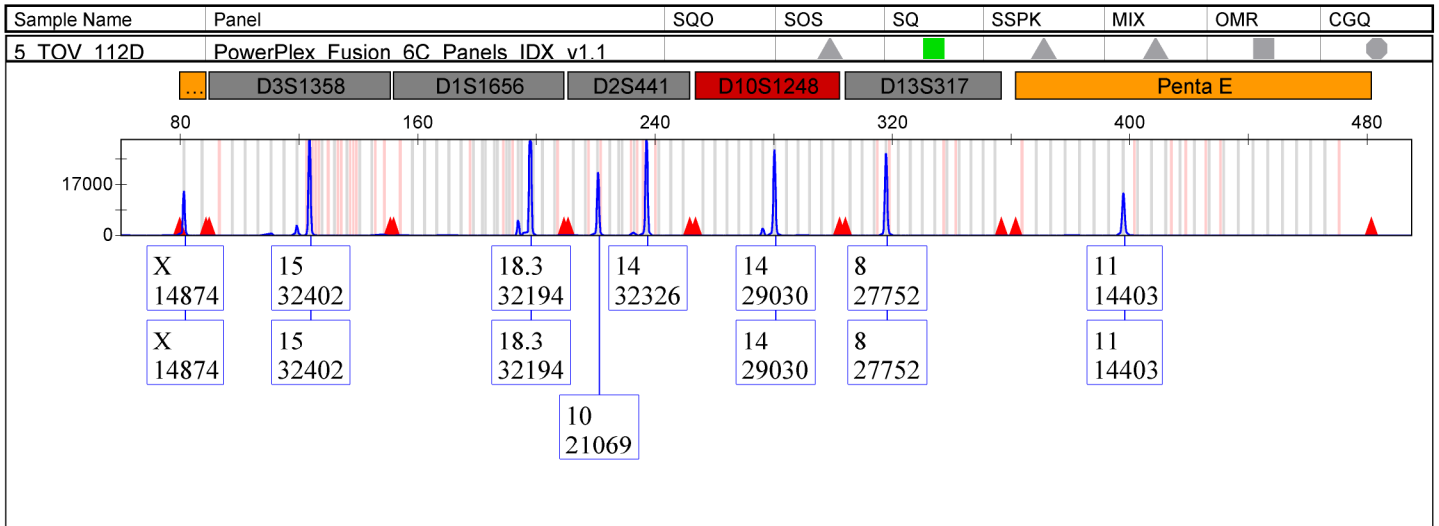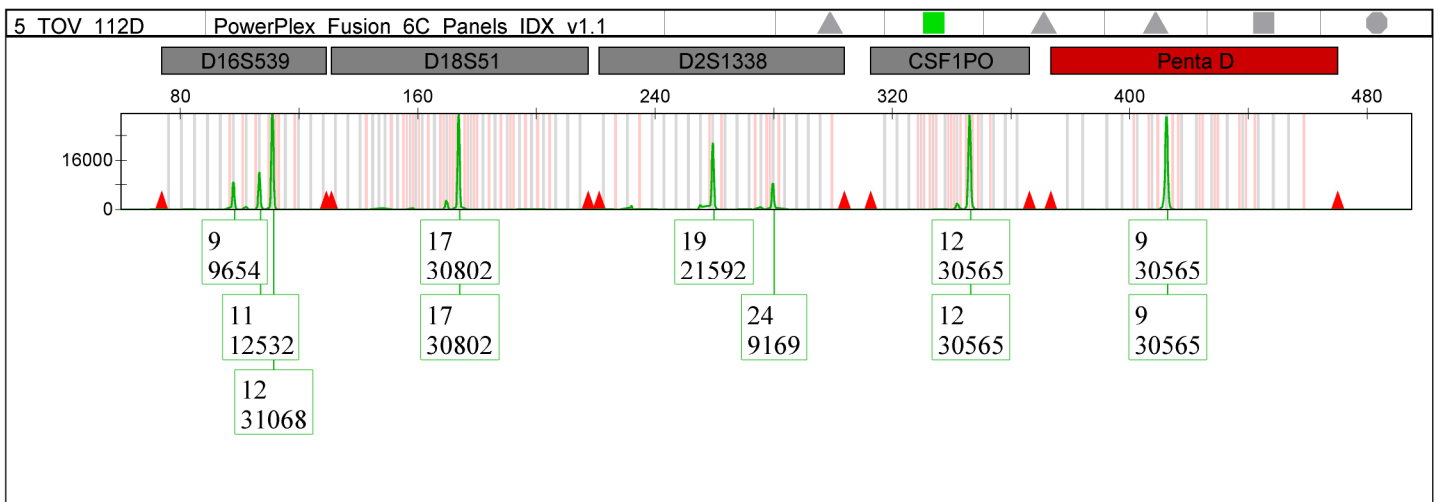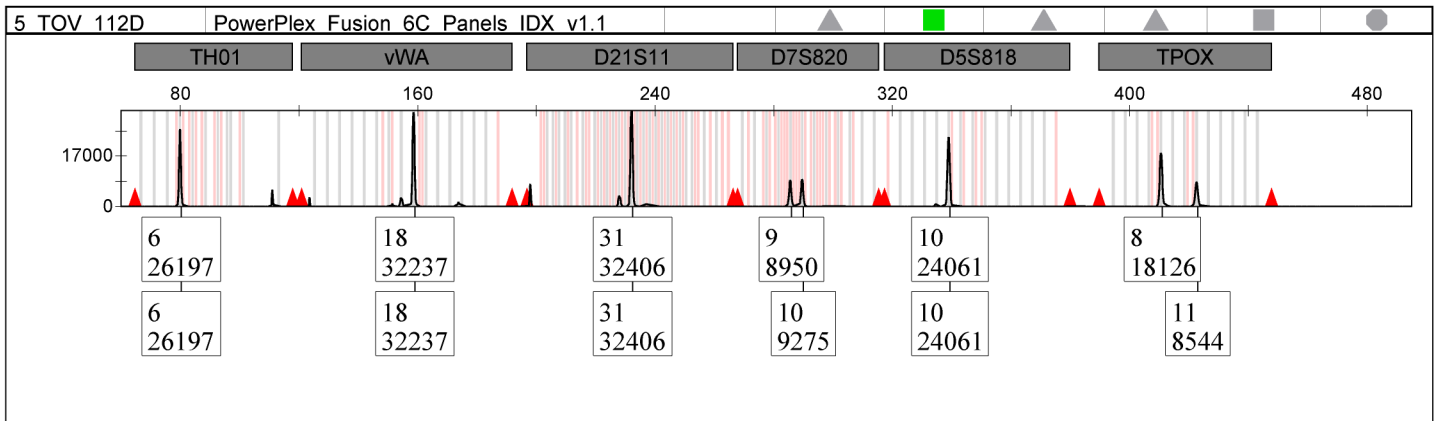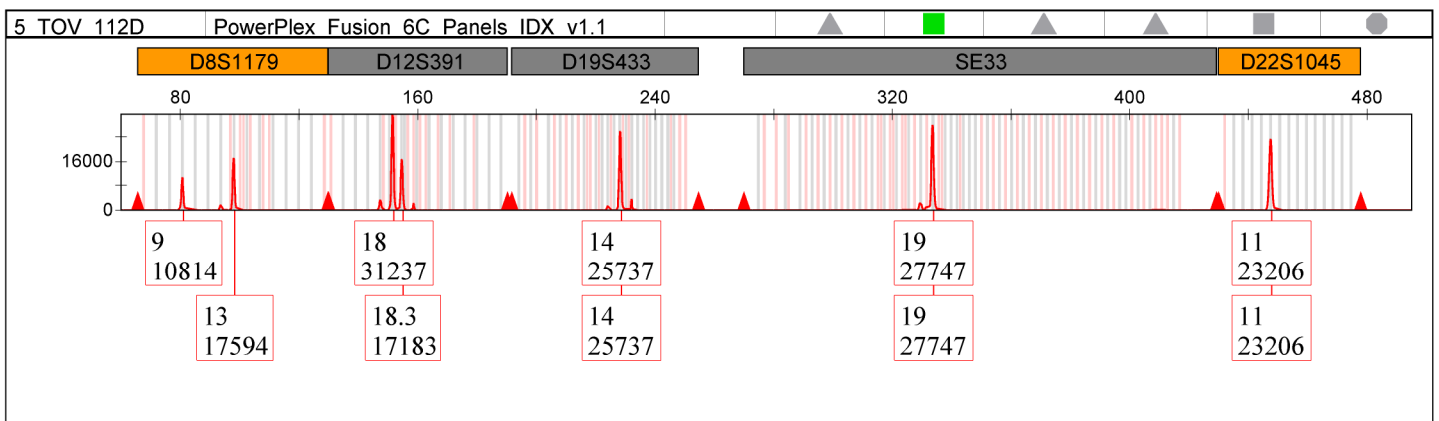

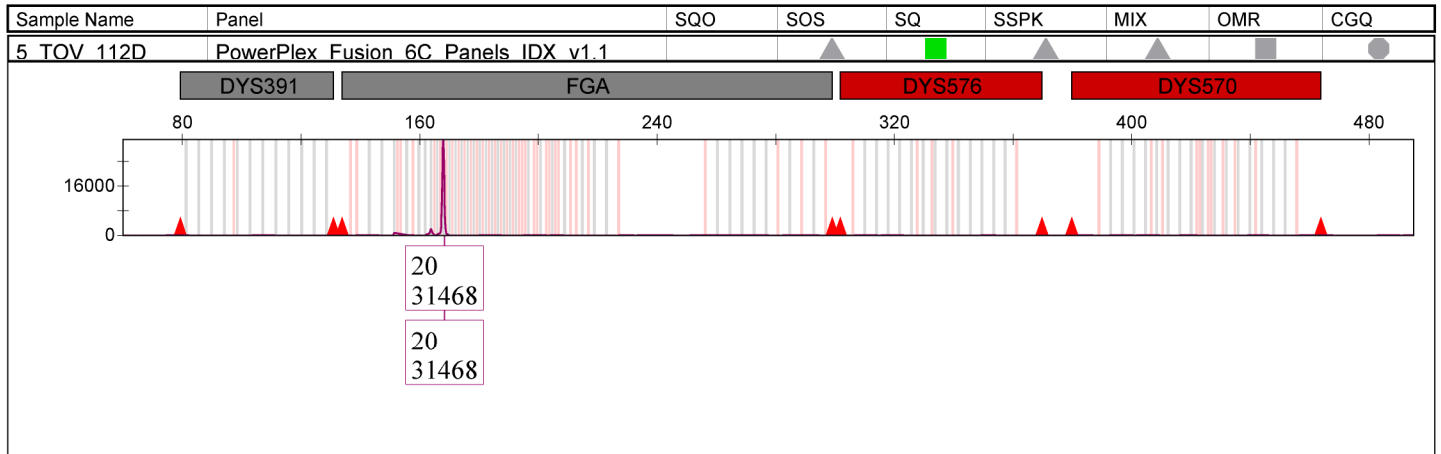

Supplement: Supplementary file 1 [file ijms-25-04381-s001.zip › Cell_lines_DNA_STR_profiling_results.pdf]
